# Monitoring insect biodiversity and comparison of sampling strategies using metabarcoding: A case study in the Yanshan Mountains, China

**DOI:** 10.1002/ece3.10031

**Published:** 2023-04-21

**Authors:** Min Li, Ting Lei, Guobin Wang, Danli Zhang, Huaxi Liu, Zhiwei Zhang

**Affiliations:** ^1^ College of Biological Science and Technology Taiyuan Normal University Jinzhong China; ^2^ Department of Life Sciences Natural History Museum London UK; ^3^ College of Forestry, Shanxi Agricultural University Jinzhong China

**Keywords:** habitat, insect biodiversity, light traps, malaise traps, metabarcoding, sweep netting

## Abstract

Insects are the richest and most diverse group of animals and yet there remains a lack, not only of systematic research into their distribution across some key regions of the planet, but of standardized sampling strategies for their study. The Yanshan Mountains, being the boundary range between the Inner Mongolian Plateau and the North China Plain, present an indispensable piece of the insect biodiversity puzzle: both requiring systematic study and offering opportunities for the development of standardized methodologies. This is the first use of DNA metabarcoding to survey the insect biodiversity of the Yanshan Mountains. The study focuses on differences of community composition among samples collected via different methods and from different habitat types. In total, 74 bulk samples were collected from five habitat types (scrubland, woodland, wetland, farmland and grassland) using three collection methods (sweep netting, Malaise traps and light traps). After DNA extraction, PCR amplification, sequencing and diversity analysis were performed, a total of 7427 Operational Taxonomic Units (OTUs) at ≥97% sequence similarity level were delimited, of which 7083 OTUs were identified as belonging to Insecta. Orthoptera, Diptera, Coleoptera and Hemiptera were found to be the dominant orders according to community composition analysis. Nonmetric multidimensional scaling (NMDS) analysis based on Bray–Curtis distances revealed highly divergent estimates of insect community composition among samples differentiated by the collection method (*R* = .524802, *p* = .001), but nonsignificant difference among samples differentiated according to habitat (*R* = .051102, *p* = .078). The study therefore appears to indicate that the concurrent use of varied collection methods is essential to the accurate monitoring of insect biodiversity.

## Graphical abstract

We successfully obtained the insect community composition of Yanshan Mountains at the order, family and genus level, using the target of *COI* gene and Illumina high‐throughput sequencing. Three collection methods (sweep netting, Malaise traps and light traps) were used to collect insects, and a total of 74 bulk samples were acquired from five habitats (scrubland, woodland, wetland, farmland and grassland). The analysis of alpha and beta diversity showed significant differences in the composition of insect communities obtained by the three different sampling methods, implying that future biodiversity assessment could be use above three collection methods concurrently.

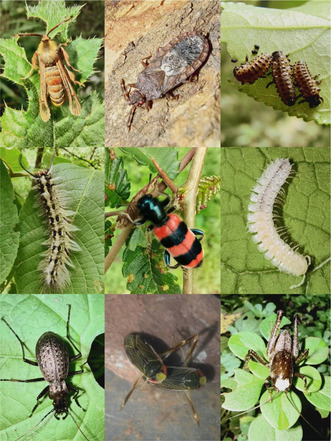

## 1 INTRODUCTION

Insects play an extremely important role in ecosystems such as pollinators, decomposers, predators and prey (Barton & Evans, 2017; Biesmeijer et al., 2006; Ganguly et al., 2022; Van Zandt et al., 2019). Because of the anthropogenic pressures, up to 1 million fauna species will be facing rapid extinction within decades (Tollefson, 2019). It is becoming increasingly urgent for us to have effective systems with which to observe and assess changes in biodiversity (Bohmann et al., 2014; Clare et al., 2022).

Historically, biodiversity monitoring has relied on the identification of species and counting of individuals by taxonomists, requiring substantial expertise, time, funding and specialization (Basset et al., 2012; Campbell et al., 2011; Wijerathna & Gunathilaka, 2020). When there is shortage of any of these, conservation scholars and environmental managers make decisions based on limited information. Given the pace of biodiversity loss, there is a need for methods of inventory that can be applied quickly and efficiently in diverse contexts, including those about which prior information is limited.

Nearly 20 years ago, the initiative of DNA barcoding offered an opportunity to identify specimens for researchers beyond the taxonomic expertise through getting a standard sequence of genes from a single sample and then searching against a reference library (Ekrem et al., 2007; Hajibabaei et al., 2007; Hebert et al., 2003, 2004). Compared with the initial approach of DNA barcoding, DNA metabarcoding makes the progress less costly but more rapidly (Braukmann et al., 2019; Valentini et al., 2016), which features it feasible to analyze the composition and structure of entire insect communities, and become a widely accepted and successfully applied in the studies of community ecology (Bittleston et al., 2022; Keck et al., 2022; Marquina et al., 2019; Piper et al., 2019; van Zinnicq Bergmann et al., 2021). The mitochondrial cytochrome c oxidase subunit I (*COI*) has the advantages of conservative, faster evolution rate and the large amount of insect taxonomic information available for this gene in the online repositories (Nehal et al., 2021; Ren et al., 2011). Therefore, *COI* locus has been the most widely used marker for metabarcoding of insects (Zhang & Hewltl, 1996).

Another historic issue facing the accurate study of insect biodiversity is the way in which methods of collection may have biases towards certain taxa. Insects samples are collected via various methods, chiefly: sweep netting, soil extraction, leaf litter collection, canopy fogging and traps (McGavin, 1997). Sweep netting is a common net type used in insect sampling which is used to capture insects flying or hovering over foliage. Malaise traps are an easily‐handled, low‐cost sampling tool which can capture flying and also wingless insects day and night. Light traps have been widely used in nocturnal insect sampling. And with robust sampling devices, light traps can collect high numbers of specimens (Yi et al., 2012). Previous studies into insect biodiversity have predominantly used one or two collection methods (Hausmann et al., 2020; Hwang et al., 2022; Kaczmarek et al., 2022; Wirta et al., 2016), and have rarely made use of three or more in combination. Different sampling methods are generally used to collect different kinds of insect taxa, with methods ordinarily chosen in accordance with the active behavior of the target (nocturnal, diurnal, low‐flying, aquatic, etc.) (McGavin, 1997; Yi et al., 2012). Two methods deployed in the same location may yield very different samples. If a sample of an area is to provide representative data on community composition, then it is key to understand how different methods of collecting will capture, miss, or otherwise proportionally affect the various taxa contained within that community.

The Yanshan Mountains are a major mountain range located to the north of the North China Plain and the south of the Inner Mongolian Plateau, containing a large number of habitats and diverse species. Positioned within one of the three largest urban agglomerations in China, containing 10 cities and with a total area of 21.7 million ha and a population of 110 million people (Cui et al., 2019; Li et al., 2020), the region is facing increasing anthropogenic pressures. Several studies have been conducted on the biodiversity of the region, including of bacteria (Tang et al., 2016), fungi (Zhou et al., 2022), flora (Bu et al., 2014; Tang et al., 2017) and large fauna (Xie et al., 2022) and arachnids (Lu et al., 2022); two have focused on insects: one on the relationship between the diversity of flower‐visiting insects and habitat types (Han et al., 2022) and another on moth communities (Hao et al., 2020).

In this study, we collected samples via three collection methods (sweep netting, Malaise traps and light traps) from five different habitats experiencing different degrees of anthropogenic impact (farmland, grassland, scrubland, wetland and woodland) and performed DNA metabarcoding. The objectives of the study were (a) to establish the insect community composition of the Yanshan Mountains through DNA metabarcoding, (b) to compare alpha diversity and beta diversity among the three collection methods and evaluate the effect of different methods on the collecting of different insect groups, and (c) to compare alpha diversity and beta diversity among different habitats and identify the dominant insect groups in the five different habitats.

## 2 MATERIALS AND METHODS

### 2.1 Sampling

**FIGURE 1:**
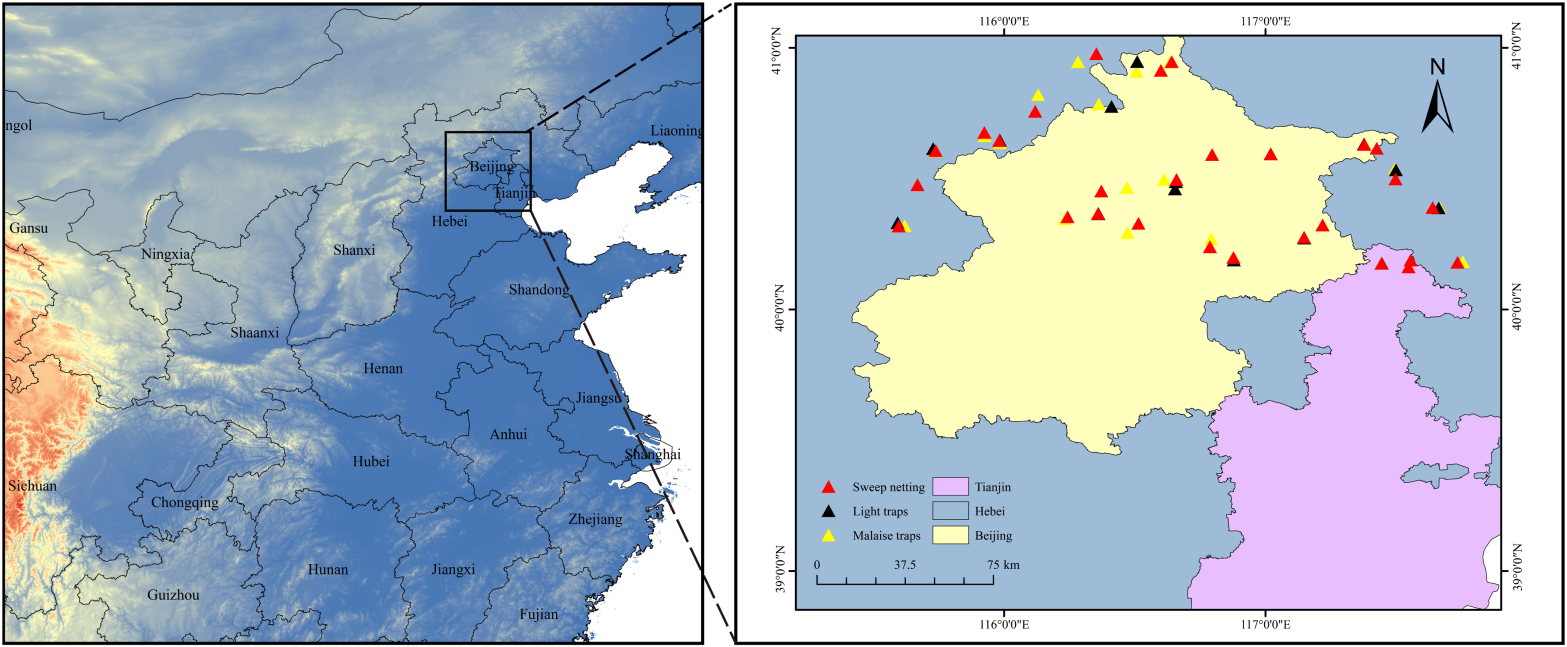
Map of sampling sites in the Yanshan Mountains. Triangles of different colors correspond to samples collected using the three different collection methods (*n* = 74, red: sweep netting; yellow: Malaise traps; black: light traps).

We divided the Yanshan Mountains into grids of 10 km^2^ and selected 30 for field sampling. To ensure inclusion of different degrees and types of anthropogenic impact, grids were categorized into habitat types (farmland, grassland, scrubland, wetland and woodland) and each type was sampled. The samples were collected via three methods: sweep netting, Malaise traps and light traps. Sweep netting and Malaise traps were set once per grid, and light traps once every two grids, due to inconvenient electricity provision and the steep terrain. All samples were collected between July and August 2019. In total, 74 samples (29 from sweep netting, 30 from Malaise traps and 15 from light traps) were obtained for the study (Figure 1; Table S1).

For the sweep netting, two sampling lines were set, each of a length of at least 200 m, and 100 sweeps performed per line. Malaise traps were left open for one each week, equipped with a jar of 99% ethanol. Light traps were run from 7:30 p.m. to 9:00 p.m. The samples were stored in 99% ethanol, except Lepidoptera specimens which were stored in triangular bags. Preservative ethanol was replaced every 24 h during collection trips (three times) and then a final time at the laboratory. The samples were then stored in a freezer at −20°C for later use.

### 2.2 Sample preparation and DNA extraction

Raw samples from each site were prepared individually by pouring the contents of the collection bottles into sterile petri dishes, sorting the insect specimens from other organisms, and then transferring them into tubes until the residual ethanol evaporated completely. To avoid the contamination from the gut content and the scales from wings, in the case of lepidopteran, specimens' wings and abdomens were removed before extraction. Specimens gathered from the same grid via the same collection method were then pooled together and treated as a single bulk sample. Each bulk sample was then ground with a sterilized pestle to homogenize the tissue. To reduce systematic errors, triplicate samples for DNA extraction were aliquoted from each bulk sample. Genomic DNA was extracted with an E‐Z 96® Mag‐Bind Soil DNA Kit in accordance with the manufacturer's protocol, and the mass and volume for each DNA extraction were checked (Table S2). A negative (no DNA template) control was processed with each extraction batch to monitor contamination.

### 2.3 PCR amplification and sequencing

The partial mitochondrial *COI* gene (313 bp) was amplified as the target region using the primers mICOIintF (5′‐GGWACWGGWTGAACWGTWTAYCCYCC‐3′) and jgHCO2198 (5′‐TANACYTCNGGRTGNCCRAARAAYCA‐3′) (Leray et al., 2013). PCR amplifications were performed in 25 μL volumes containing the following: 5 × reaction buffer 5 μL; 5 × GC buffer 5 μL; dNTP (2.5 mM) 2 μL; Forward primer (10 μM) 1 μL; Reverse primer (10 μM) 1 μL; DNA Template 2 μL; ddH2O 8.75 μL; Q5 DNA Polymerase 0.25 μL. The PCR protocol comprised: initial denaturation (98°C for 2 min); 25–30 cycles of denaturation (98°C for 15 s), annealing (55°C for 30 s) and extension (72°C for 30 s); final extension (72°C for 5 min). Primers were given different unique tags for each amplification. Amplification results were checked using gel electrophoresis on 1.20% agarose gel. The target amplicons were purified with the AxyPrep DNA Gel Extraction Kit (Axygen) following the manufacturer's instructions. The purified amplicons of triplicates subsamples were pooled together for library preparation. Sequencing libraries were prepared using Illumina's TruSeq Nano DNA LT Library Prep Kit. Ligated PCR products were purified using BECKMAN AMPure XP beads. Sequencing was carried out using the Illumina MiSeq PE300 platform (Personalbio).

### 2.4 Bioinformatics and data processing

Sequence processing was performed with cutadapt v2.3 (Martin, 2011) and vsearch v2.13.4 suite (including fastq_mergepairs, fastq_filter, derep_fulllength, cluster_size and uchime_denovo) (Rognes et al., 2016). Initial quality control was carried out with fastQC. Cutadapt v2.3 was used to trim primers and remove sequences with unmatched primers. Pair‐merging was performed using fastq_mergepairs with 202 bp overlap. Quality filtering was performed using fastq_filter, with a maximum expected error of 0.5. Derep_fulllength was used for dereplicating. Uchime_denovo was used to further remove the chimeras. High‐quality sequences were then clustered into operational taxonomic units (OTUs) using cluster_size with a sequence identity threshold of 97% (Creedy et al., 2019; Meier et al., 2006). Singletons of all specimens were removed, and the complete OTU table generated.

### 2.5 Statistical analysis

For data obtained from NCBI contained more unique genus and species labels than BOLD arthropod references (Robeson et al., 2021), the representative sequences of each OTU were blasted against the nt database (ftp://ftp.ncbi.nih.gov/blast/db/) with “blastn” (Camacho et al., 2009). The complete OTU table was then analyzed using QIIME2 (Bolyen et al., 2019) to reveal the community composition both of each individual sample and of the Yanshan Mountains community taken as a whole. The OTUs identified as not insects were then removed. Rarefication of all samples was performed to check the sequencing depth (Heck et al., 1975). Using QIIME2, all samples were rarefied with a threshold set to 95% of the number of sequences of the sample with the lowest count (19,117). The insect OTU table was then generated and the rarefaction curves were obtained.

Venn diagram and biodiversity (alpha and beta) analyses based on the insect OTU table were carried out in R v3.6 (R Development Core Team, 2017). The Venn diagrams were generated in VennDiagram (Chen & Boutros, 2011). The alpha diversity indices (Chao1, Good's coverage, Pielou's evenness, Shannon and Simpson) were estimated with ggplot2 (Ginestet, 2011). Chao1 is used for estimating the number of species actually present in the insect community. Good's coverage for the proportion of non‐singleton OTUs, Pielou's evenness for the evenness, and both Shannon and Simpson for the diversity. The results of the five alpha diversity indices were subjected to Kruskal‐Wallis tests (one for each of the collection‐method groups) (Theodorsson‐Norheim, 1986) and Dunn's tests (one for each pairwise comparison) (Dinno, 2017). Differences in OTU composition among collection methods and habitat types were examined using non‐metric multidimensional scaling (NMDS) based on Bray–Curtis and Jaccard dissimilarity matrices in vegan (Dixon, 2003); analysis of similarities (ANOSIM) tests were also applied using 999 permutations. Heatmaps were produced using pheatmap (Kolde, 2019).

## 3 RESULTS

### 3.1 Sequencing summary and OTU delimitation

A total of 3,685,110 raw reads were produced. After quality filtering and chimera detection, 3,471,955 were retained; after filtering singletons 3,468,592; after length filtering 3,250,555 (Table S3). A total of 7427 OTUs were clustered from all samples (Table S4), of which 98.47% (7313) were identified against the nt database, and 95.37% (7083) were identified to insects. Of the insect OTUs, 25.68% (1819) were assigned to species level. After removing non‐insect OTUs, 4710 were obtained from sweep netting, 3085 from Malaise traps and 978 OTUs from light traps. Organizing by habitat type, 4443 were obtained from scrubland; 3305 from woodland; 1990 from wetland; 1304 from farmland and 310 from grassland. The five highest numbers of OTUs were obtained from sweep netting 1 (S1) (832), S3 (956), S4 (864), S7 (1079) and S8 (610); light traps 11 (LT11) (40) and LT15 (44) were the two samples containing the lowest numbers.

### 3.2 Community composition at different taxonomic levels

#### 3.2.1 Order level

**FIGURE 2:**
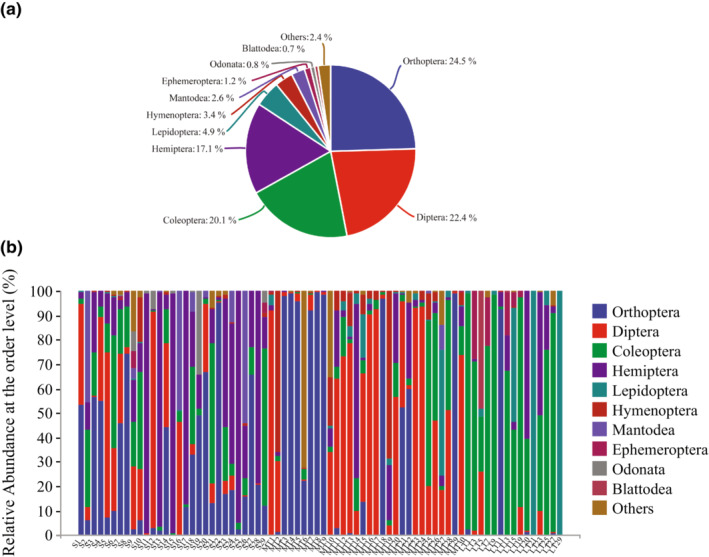
Statistics showing insect community composition at order level. (a) Main orders detected in the Yanshan Mountains. (b) Community composition of each sample. For the legend on the right, from top to bottom, the abundance of the orders was ranked from most to least.

Apart from 74 OTUs that were not identified at the order level, all insect OTUs were classified into 16 orders. Order level community composition diagrams of each sample and for the whole Yanshan Mountains community were generated (Figure 2). Considering the whole Yanshan Mountains samples as a whole, Orthoptera (24.5%) occupied the highest portion, followed by Diptera (22.4%), Coleoptera (20.1%), Hemiptera (17.1%), Lepidoptera (4.9%), Hymenoptera (3.4%), Mantodea (2.6%), Ephemeroptera (1.2%), Odonata (0.8%), and others (2.4%) (Figure 2a). Species richness for the most part corresponded to abundance, with OTUs obtained as follows: Orthoptera 3320; Diptera 1180; Coleoptera 982, Hemiptera 843, Lepidoptera 251, Hymenoptera 271, Mantodea 60, Ephemeroptera 57 and Odonata15.

Almost half (35 of 74) of the samples can be categorized into for lists according to how each is dominated by a single order, with the order in question accounting for more than 70% of each sample in each list. Orthoptera dominated the samples S8, S22, Malaise traps 3 (MT3), MT4, MT5, MT7, MT8, MT9, MT18, MT29 and LT6; Diptera dominated S12, MT1, MT12, MT13, MT16, MT17, MT23, MT24 and MT30; Coleoptera dominated LT1, LT2, LT4, LT5, LT7, LT9, LT13 and LT14; Hemiptera dominated S11, S13, S15, S17, S25, S28 and MT14 (Figure 2b).

#### 3.2.2 Family level

Family‐level community composition diagrams for each sample and the whole Yanshan Mountains community were generated (Figure S1). Tettigoniidae (Orthoptera) (14.1%) was the most dominant, followed by Scarabaeidae (Coleoptera) (11.1%), Acrididae (Orthoptera) (8.1%), Asilidae (Diptera) (5.1%), Dolichopodidae (Diptera) (5.1%), Cicadidae (Hemiptera) (5.1%), Fulgoridae (Hemiptera) (4.0%), Chrysomelidae (Coleoptera) (3.0%), Formicidae (Hymenoptera) (2.0%) and others (39.4%) (Figure S1a). There was no significant correspondence between species richness and abundance, with OTUs obtained as follows: Acrididae 2324, Tettigoniidae 840, Chrysomelidae 327, Scarabaeidae 235, Dolichopodidae 115, Fulgoridae 98, Cicadidae 67, Formicidae 65 and Asilidae 45.

Over a quarter (20 of 74) of the samples can be categorized into three lists according to how each is dominated by a single family, with the family in question accounting for more than 70% of each sample in two of the lists, and more than 50% in the third: Tettigoniidae (70%) dominated the samples S22, MT3, MT4, MT5, MT7, MT8, MT9, MT18 and MT29; Scarabaeidae (70%) dominated the samples LT1, LT4, LT5, LT13 and LT14, Acrididae (50%) dominated the samples S1, S3, S4, S20, S27 and MT22 (Figure S1b).

#### 3.2.3 Genus level

No significant findings were made at genus level. Genus‐level community composition diagrams for each sample and for the whole Yanshan Mountains were generated (Figure S2). *Atlanticus* (9.9%) was the most dominant, followed by *Lycorma* (4.0%), *Hyalessa* (4.0%), *Melolontha* (3.0%), *Anomala* (3.0%), *Machimus* (3.0%), *Amblypsilopus* (3.0%), *Chorthippus* (2.0%), *Cletus* (2.0%), *Medetera* (2.0%) and others (64.4%) (Figure S2a). There was no correspondence between species richness and abundance, with OTUs obtained as follows: *Chorthippus* 987, *Atlanticus* 411, *Lycorma* 86, *Cletus* 42, *Anomala* 38, *Medetera* 31, *Hyalessa* 29, *Melolontha* 23, *Machimus* 20 and *Amblypsilopus* 3.

Fewer than a fifth (14 of 74) of the samples can be categorized into lists according to how each is dominated by a single genus: *Atlanticus* (>70%) dominated the samples S22, MT3, MT4, MT5, MT7, MT8, MT9 and MT29; *Lycorma* (>90%) dominated the samples S12 and S25; accounting for more than 90% of the sample itself. *Hyalessa* (>60%) dominated the samples S1, S15, S17 and LT12 (Figure S2b).

### 3.3 Alpha diversity analyses

After rarefying, 6447 OTUs were obtained (Table S5). Rarefaction curves were constructed for each individual sample (Figure S3). The rarefaction curves of 70 out of 74 samples reached the saturation stage. Diversity indices (Chao1, Good's coverage, Pielou's evenness, Shannon and Simpson) were calculated with ggplot2 (Table S6): The Chao1 index showed the highest richness of species to be in S7, S3 and S1 and the lowest in S16, LT21 and LT29; Good's coverage showed that high coverage (>98%) was achieved in samples; Pielou's evenness showed S7 to have the greatest evenness and LT6 to have the least; Simpson and Shannon diversity indices showed the insect biodiversity to be highest in S7 and lowest in LT6.

### 3.4 Group comparison analyses

#### 3.4.1 Comparison for samples collected by different methods

The number of insect OTUs detected was highest in the combined samples collected using sweep netting (4100), followed by Malaise traps (2728) and light traps (859), shown in the Venn diagram (Figure S4). The numbers of OTUs unique to each were 3096, 1718 and 537, respectively. The OTUs shared between collection‐method groups were as follows: sweep netting with Malaise traps 774; sweep netting with light traps 86; Malaise traps with light traps; all three 144.

Order‐level analysis of community composition showed more Hemiptera collected from sweep netting than from Malaise or light traps, more Diptera and Orthoptera collected from Malaise traps than from light traps or sweep netting, and more Coleoptera and Lepidoptera collected from light traps than from Malaise traps or sweep netting. Order‐level analysis identified 27 families appearing only in the sweep netting group, 45 only families in the Malaise traps group, and 19 only in the light trap group (Table S7).

**FIGURE 3:**
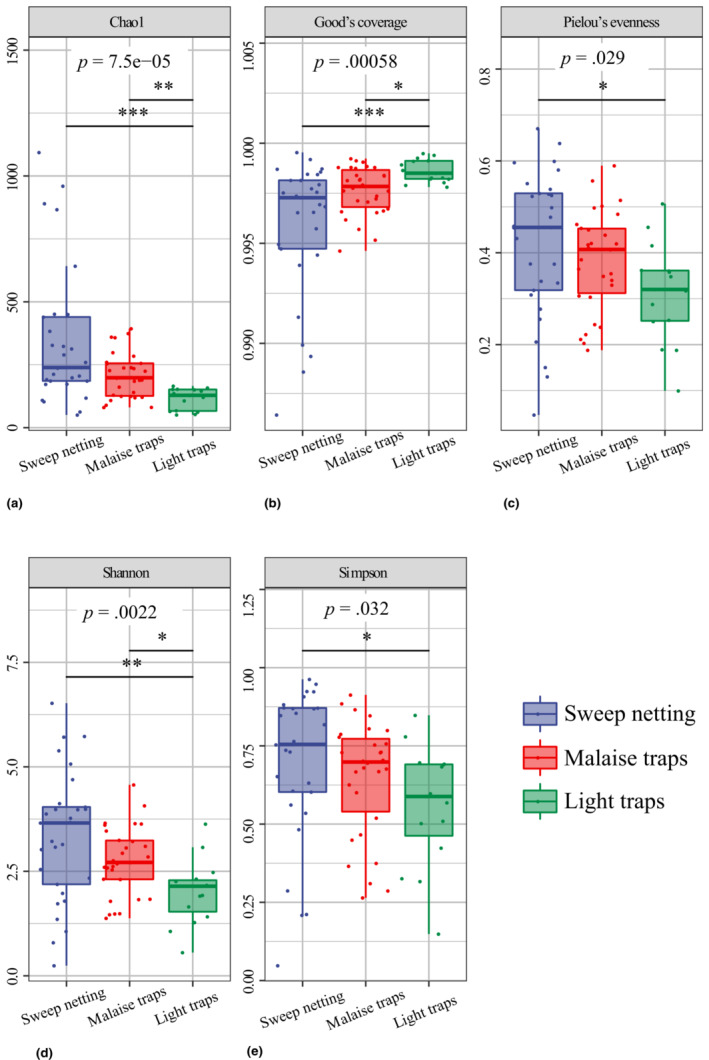
Alpha diversity estimates of the three collection‐method groups. Box plots display the first and third quartiles and the median, maximum and minimum observed values within each dataset. The number under each diversity index label is the *p*‐value of the Kruskal‐Wallis test. The short line represents the significant difference between different collection‐method groups calculated using Dunn's test. * represents the degree of difference: *p < .05, **p < .01, ***p < .001.

The results of the five alpha diversity indices, Kruskal‐Wallis test, and Dunn's test are displayed in Figure 3. Chao1 showed species richness to be the lowest in the light traps group (Figure 3a); Good's coverage that the light traps group had the highest proportion of detected species (Figure 3b); Pielou's evenness that the sweep netting group had the highest evenness value (Figure 3c); and both Shannon and Simpson that the light traps group had the lowest diversity (Figure 3d,e). The Kruskal‐Wallis tests for each alpha diversity index showed significant difference among the three collection‐method groups. The Dunn's tests showed significant difference either between the light traps group and both other groups, or between the light trap group and the sweep netting group.

**FIGURE 4:**
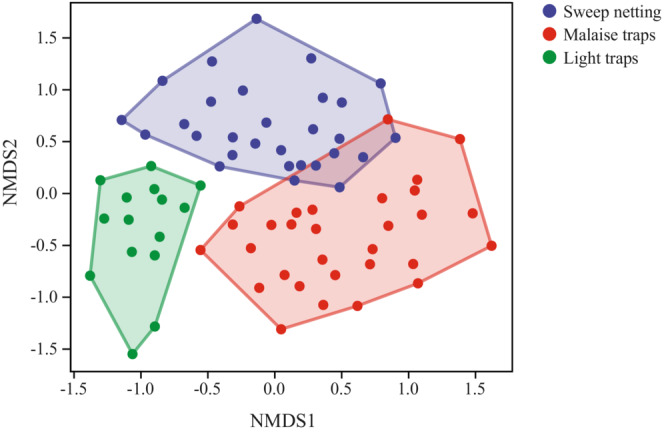
NMDS analysis of insect community similarity among the different collection‐method groups based on a Bray–Curtis dissimilarity matrix.

NMDS plots based on Bray–Curtis and Jaccard distances clear clustering of samples from each collection‐method group and significant differences among different collection‐method groups (Figure 4 and Figure S5 respectively). The result of ANOSIM tests indicated that insect community composition differs significantly among collection‐method groups (*R* = .413878, *p* = .001). The pairwise differences were as follows: sweep netting group and Malaise trap group (*R* = .312928, *p* = .001); sweep netting group and the light traps group (*R* = .498392, *p* = .001); Malaise traps and light traps (*R* = .524802, *p* = .001).

Heat maps of all the OTUs that could be identified to order or family level are displayed in their collection‐method groups in Figure S6. At order level: Blattodea dominated the light traps group, Odonata the sweep netting group and Trichoptera the Malaise trap group (Figure S6a); at family level, Corydiidae dominated the light traps group, Libellulidae the sweep netting group and Ceratopogonidae the Malaise trap group (Figure S6b).

#### 3.4.2 Comparison of samples collected from different habitat types

**FIGURE 5:**
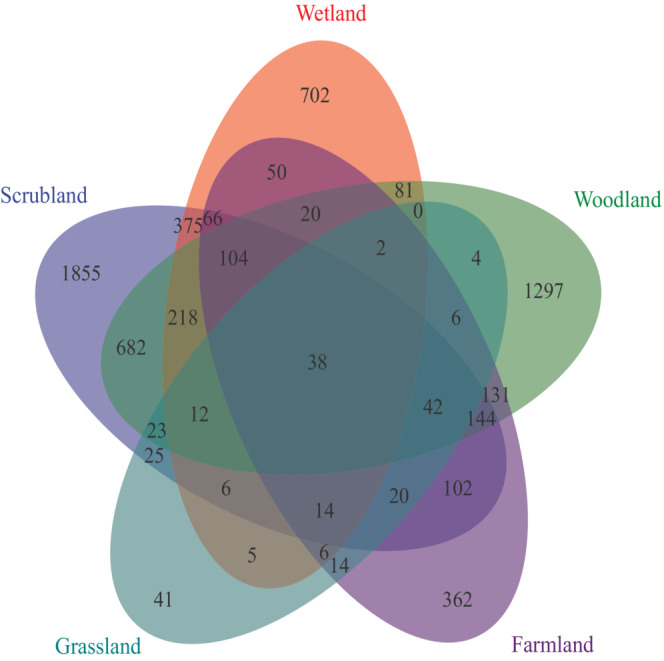
Number of shared and unique OTUs across the five different habitats.

The number of insect OTUs detected was highest in the combined samples collected from scrubland (3726), followed by woodland (2804), wetland (1699), farmland (1121) and grassland (258), shown in the Venn diagram (Figure 5). The numbers of OTUs unique to each were 1855, 1297, 702, 362 and 41, respectively. The numbers of families detected exclusively in each habitat type were as follows: woodland 25, scrubland 21, wetland 7, farmland 6, grassland 0 (Table S8).

The results of the five alpha diversity indices, Kruskal‐Wallis tests, and Dunn's tests are displayed in Figure S7. The Kruskal‐Wallis tests for four of the alpha diversity indices showed significant difference among the five habitat types (Chao1 *p* = .0076; Good's coverage *p* = .013; Pielou's evenness *p* = .047; Shannon *p* = .019) (Figure S7a–d), with the exception of Simpson, which showed nonsignificant difference (*p* = .098) (Figure S7e). Dunn's tests showed that species richness in the wetland samples was significantly higher than in the farmland samples (*p* = .03).

NMDS analysis based on Bray–Curtis index of the recovered communities from the different habitats under the same collection method were shown in Figure S8. NMDS plots based on Bray–Curtis distances displayed nonsignificant difference among the samples from five different habitat types when collected via sweep netting (Figure S8a); Furthermore, NMDS plots based on Bray–Curtis distances displayed similarities among the samples recovered from wetland, woodland, scrubland and farmland habitats via Malaise traps (Figure S8b), and among the samples recovered from woodland, scrubland and farmland habitats via light traps (Figure S8c). NMDS plots based on Jaccard distances displayed nonsignificant difference among the samples from different habitat types when collected via the same method (Figure S9). ANOSIM tests indicated similarities among the samples from different habitat types (*R* = .051102, *p* = .078) and nonsignificant pairwise differences between habitats (Table S9).

**FIGURE 6:**
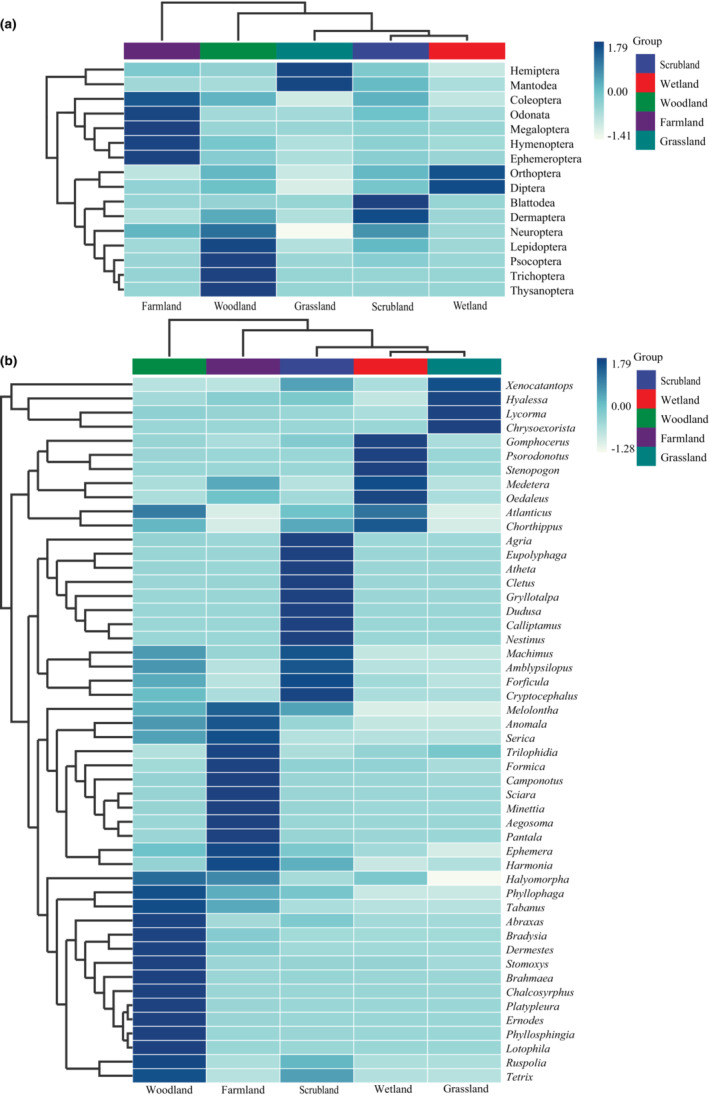
Differential abundance heatmap of samples comparing the five different habitat types. (a) Heat map showing the abundance of the top 16 orders; (b) Heat map showing the abundance of the top 50 genera.

Heat maps of all the OTUs that could be identified to order or genus level are displayed according to their habitat types in Figure 6. Large variations exist in the species detected across all the samples. At order level, high abundance was identified in habitat types as follows: Blattodea and Dermaptera in scrubland; Neuroptera, Lepidoptera, Psocoptera, Trichoptera and Thysanoptera in woodland; Orthoptera and Diptera in wetwood; Coleoptera, Odonata, Megaloptera, Hymenoptera and Ephemeroptera in farmland; Hemiptera and Mantodea in grassland (Figure 6a). At genus level, *Nestinus* dominated scrubland, *Lotophila* woodland, *Stenopogon* wetland, *Aegosoma* farmland, and *Chrysoexorista* grassland (Figure 6b).

## 4 DISCUSSION

We completed a project of large‐scale bio‐monitoring for the Yanshan Mountains, using a 313 bp fragment of the partial *COI* gene and Illumina high‐throughput sequencing, illustrating how different collection methods may produce different characterizations of a single community. It is clear that the methods of collection had biases towards certain taxa in our study. If bio‐monitoring is to be effective, then understanding how various collection methods may affect the composition of the resulting samples is key to the future study of insect biodiversity. Although metabarcoding is indeed a promising approach for the study of global biodiversity (Cristescu, 2014; Zhang et al., 2022), a comprehensive understanding of sampling methodology is central to its future accuracy.

### 4.1 Samples preparation and PCR amplification

It is thought that the relative read abundance is influenced by methodological issues than by ecological issues (Deagle et al., 2019; Piñol et al., 2019). In a study of complex arthropod communities, Creedy et al. (2019) concluded that OTU recovery was not skewed by variations in the biomass of the constituent species, as long as sequencing depth was sufficient and specimens were within reasonable size ranges. However, they point out that all primers target specific taxa, and that therefor in any PCR‐based study of community composition, choice of primer will have an inevitable influence on results: the biases introduced by choices of primer are therefore likely to affect the evaluation of species richness, but not of turnover. In future biodiversity studies, these limitations may be overcome to some degree by the use of, single or in combination: PCR‐free library construction, single‐molecule real‐time sequencing, and strategies based on combining multiple sets of primers.

The ecological and biological issues might also introduce bias for the relative read abundance. In this study, the community composition results demonstrated that the dominant order, family and genus were Orthoptera, Tettigoniidae and *Atlanticus*, respectively. One reason for that may be Orthopterans occupied relative larger individual can result in more similar read numbers than many small ones. Sorting bulk samples by specimen biomass or body size could improve taxa detection using DNA metabarcoding (Elbrecht et al., 2017). However, we chose the current method as it makes less susceptible to contamination, and the purpose of the study was not to detect small and rare taxa in the bulk samples, which were also discussed by Elbrecht et al. and other researchers (Aylagas et al., 2016; Klunder et al., 2022).

### 4.2 Molecular biodiversity assessment and community composition analysis

In our study, most samples reached saturation, indicating that the sequencing depth was appropriate for diversity analyses. Most (98.47% of 7427) OTUs were identified against the nt database; a reason for the extant unidentified OTUs could be a lack of the corresponding barcodes in the reference databases. This might be offered as an explanation for the low number of insect OTUs assigned to species level (1819, or 25.68%). These low numbers underline the lack of existing information about insect biodiversity in Yanshan Mountains, and illustrate the need for comprehensive, well‐curated reference database in future biodiversity research.

A small number of OTUs (344, or 4.63%) were identified as non‐insect, and included bacteria, fungi, himatismenida and oomycetes. One possible interpretation could be that these OTUs were attached to or had been ingested by the insect specimens. It is also possible that the OTUs identified as being bacteria are actually errors that come from the blastn process, misidentified as a result of too‐ short fragments and insufficient database references. Recent studies have shown that the primers used in this study are capable of amplifying fungi DNA, even though they were primarily designed for metazoan (Leray & Knowlton, 2015; Zenker et al., 2020).

The results of community composition analyses at order level indicate a correlation between abundance and richness the rankings, although at the family and genus levels, no such correlation was noted. If the correlation at order‐level between richness and abundance is a meaningful finding rather than an anomaly, it can probably only be illuminated through a further and more detailed analysis of the insect biodiversity of the Yanshan Mountains.

### 4.3 Collection of samples collected via different collection methods

In our study, species richness was the highest in the samples obtained via sweep netting, followed by Malaise traps and light traps respectively. Reasons for differences in species richness between collection methods can be hypothesized, but as with some other aspects of this initial study, only further research can form the basis for anything more solid than speculation. One possible explanation may be that suggested by Yi et al. (2012), which is that methods of collection that minimize tailoring to specific behavior and attractants may result in catches that containing higher richness than other, more tailored, forms of capture. They categorize sampling methods into three groups: the least biased being passive sampling methods without any “activity density” bias, including sweep netting; passive sampling methods with “activity density” bias, including Malaise traps, and active sampling methods, including light traps.

Different collection methods indicated different taxa biases. Hemiptera was detected at a greater abundance in samples obtained by sweep netting than by Malaise traps or light traps: a possible reason for this might be that sweep netting in our study was focused on weeds and the dorsal sides of leaves, places where hemipteran insects often stay during daytime. Malaise traps resulted in the highest numbers of Diptera and Orthoptera, consistent with previous findings that Malaise traps are suitable for capturing airborne insects with high levels of mobility (Beng et al., 2016; Huang et al., 2022). Due to inconvenient electricity provision and the steep terrain, light traps were performed 15 times in this study. Light traps obtained the highest numbers of Coleoptera and Lepidoptera, consistent with previous indications that members of these orders are often easily attracted by artificial light sources (Nag & Nath, 2010; Thein & Choi, 2016). These differences among the specimens obtained via alternative collection methods were underlined by our finding that differences in community composition among the three collection methods were both significant and greater than those within each group.

The use of all three collection methods resulted in obtaining of specimens that would not have been gathered using only two. Although sweep netting recovered more OTUs (3096) than the other two collection methods combined (Malaise traps 1718; light traps 537), the alternative methods were mutually complimentary, illustrated by the limited overlap of 144 OTUs.

### 4.4 Comparison of samples collected from different habitat types

Alpha diversity indices showed that species richness, community evenness and diversity were highest in the wetland habitats and lowest in grassland, indicating that the community structure is most stable in the former and least stable in the latter. The stability of insect community structure in farmland and grassland appears to both be relatively weak and therefore present cause for concern. Further investigation of the insect communities is therefore warranted for the purpose of formulating interventions to promote biodiversity in the habitat types.

The habitats of the collection sites included five types: scrubland, woodland, wetland, farmland and grassland. In accordance with previous studies of insect biodiversity surveying different habitats (Beng et al., 2016; Dopheide et al., 2020; Phalan et al., 2011), we expected significant differences of community composition among samples from different habitat types. However, NMDS ordinations displayed similarities in the composition of communities from different habitat types obtained via the same collection methods, and some overlap among all samples from these five habitat types (*R* = .051102, *p* = .078). Where results indicated low beta diversity, we can surmise that the habitats in question are likely to have relatively similar compositions of species. The lack of differences among the five habitat types may be due to, firstly, the low number of replicates for each habitat type, and secondly, comparative similarities of the different habitats.

The species composition heatmaps showed significant differences in dominant order or genus among the five different habitat types, indicating large variations in insect community composition among them. The heatmap results showed high abundance of Hymenoptera (along with Coleoptera, Odonata, Megaloptera and Ephemeroptera) in the farmland. Since many species of Hymenoptera are pollinators or parasites, we might hypothesize that this is because the farmland may be supplying them with pollinating flowers and prey.

## 5 CONCLUSIONS

In this study, we established initial insect community compositions of the Yanshan Mountains and conducted comparison of three collection methods and five different habitats. Based on our results, we confirm that DNA metabarcoding provides a cost‐ and time‐efficient way to recover information on the species composition of insect communities. However, the collection methods, crucial for final DNA capture, remain both unstandardized and incompletely understood. The differences we observed among samples collected using different methods indicate poorly‐understood factors in the obtaining of bio‐monitoring data. A more comprehensive understanding of sampling methodology is therefore central to the future accuracy and efficacy of metabarcoding, particularly in the field of biodiversity analysis.

## AUTHOR CONTRIBUTIONS

**Min Li:** Conceptualization (lead); data curation (supporting); funding acquisition (equal); methodology (equal); resources (equal); supervision (lead); writing – original draft (supporting); writing – review and editing (lead). **Ting Lei:** Data curation (lead); investigation (lead); methodology (equal); resources (supporting); software (lead); visualization (equal); writing – original draft (equal); writing – review and editing (equal). **Guobin Wang:** Data curation (supporting); methodology (supporting); resources (supporting); software (supporting); visualization (lead). **Danli Zhang:** Data curation (equal); investigation (supporting); methodology (equal); software (equal); supervision (equal); validation (equal). **Huaxi Liu:** Conceptualization (equal); formal analysis (lead); methodology (supporting); supervision (equal); validation (lead); writing – review and editing (lead). **Zhiwei Zhang:** Conceptualization (lead); funding acquisition (lead); investigation (equal); project administration (lead); supervision (equal); validation (equal); writing – review and editing (equal).

## CONFLICT OF INTEREST STATEMENT

The authors declare that they have no known competing financial interests or personal relationships that could have appeared to influence the work reported in this paper.

### OPEN RESEARCH BADGES

This article has earned an Open Data badge for making publicly available the digitally‐shareable data necessary to reproduce the reported results. The data is available at https://doi.org/10.5061/dryad.x3ffbg7p7.

## Supporting information


Figure S1

Figure S2

Figure S3

Figure S4

Figure S5

Figure S6

Figure S7

Figure S8

Figure S9

Table S1

Table S2

Table S3

Table S4

Table S5

Table S6

Table S7

Table S8

Table S9

## Data Availability

The raw data with the DNA sequences presented in this study are under Bioproject PRJNA804856, BioSample accession SAMN25810026‐SAMN25810099 and NCBI SRA: SRR17956399‐SRR17956326. Supplementary materials could be obtained from Dryad with https://doi.org/10.5061/dryad.x3ffbg7p7.
