# Supplementary material for: Monitoring insect biodiversity and comparison of sampling strategies using metabarcoding: A case study in the Yanshan Mountains, China
Source: Ecol Evol. 2023 Apr 21;13(4):e10031. doi: 10.1002/ece3.10031 (PMC10121320; doi:10.1002/ece3.10031)

**FIGURE S1** Statistics of insect community composition at the family level. (**a**) Proportion of main families for Yanshan Mountains was provided in pie chart. (**b**) The community composition diagram for each sample at the family level. For the legend on the right, from top to bottom, the abundance of the families was ranked from most to least.


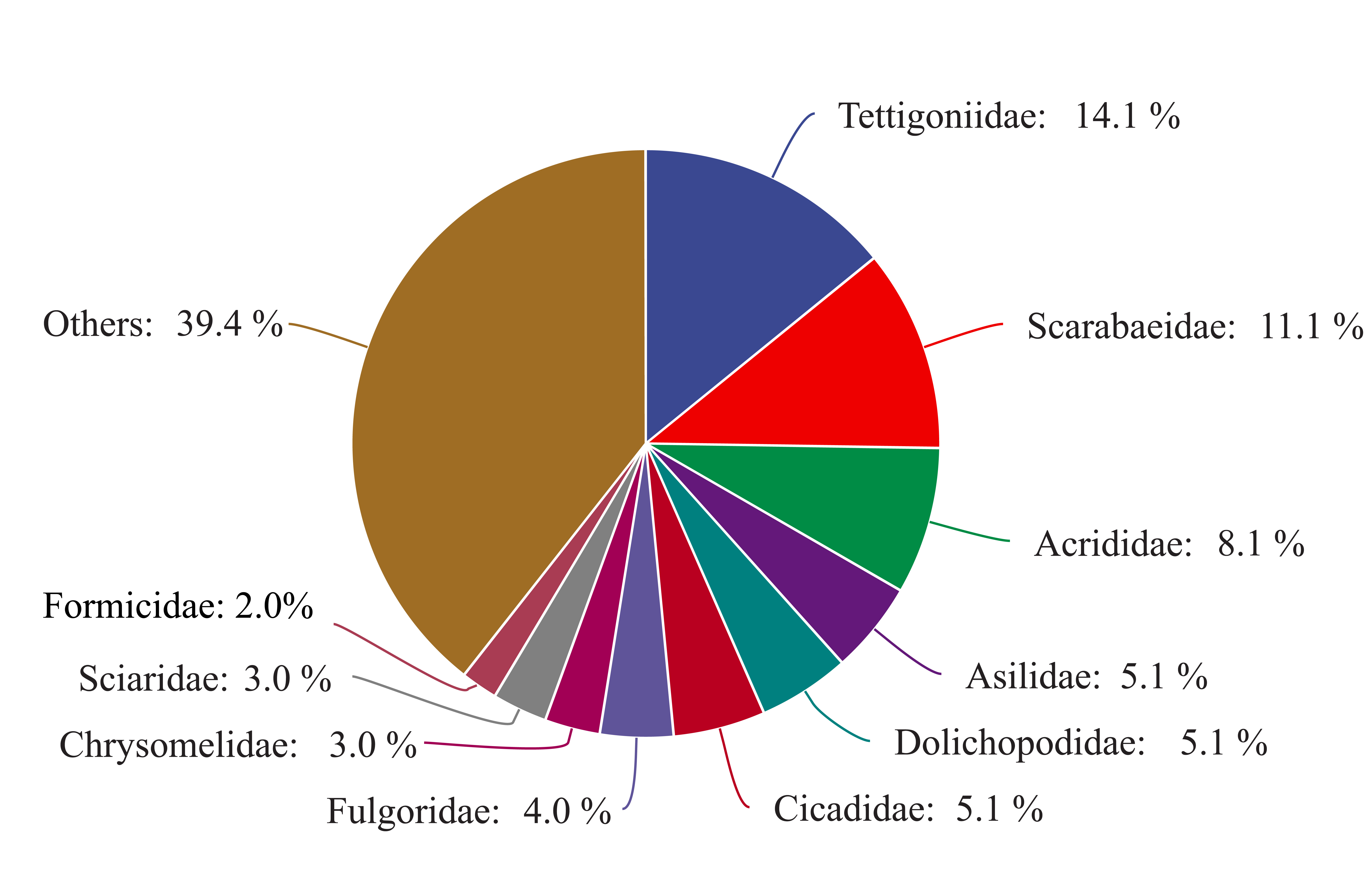


(a)

(b)


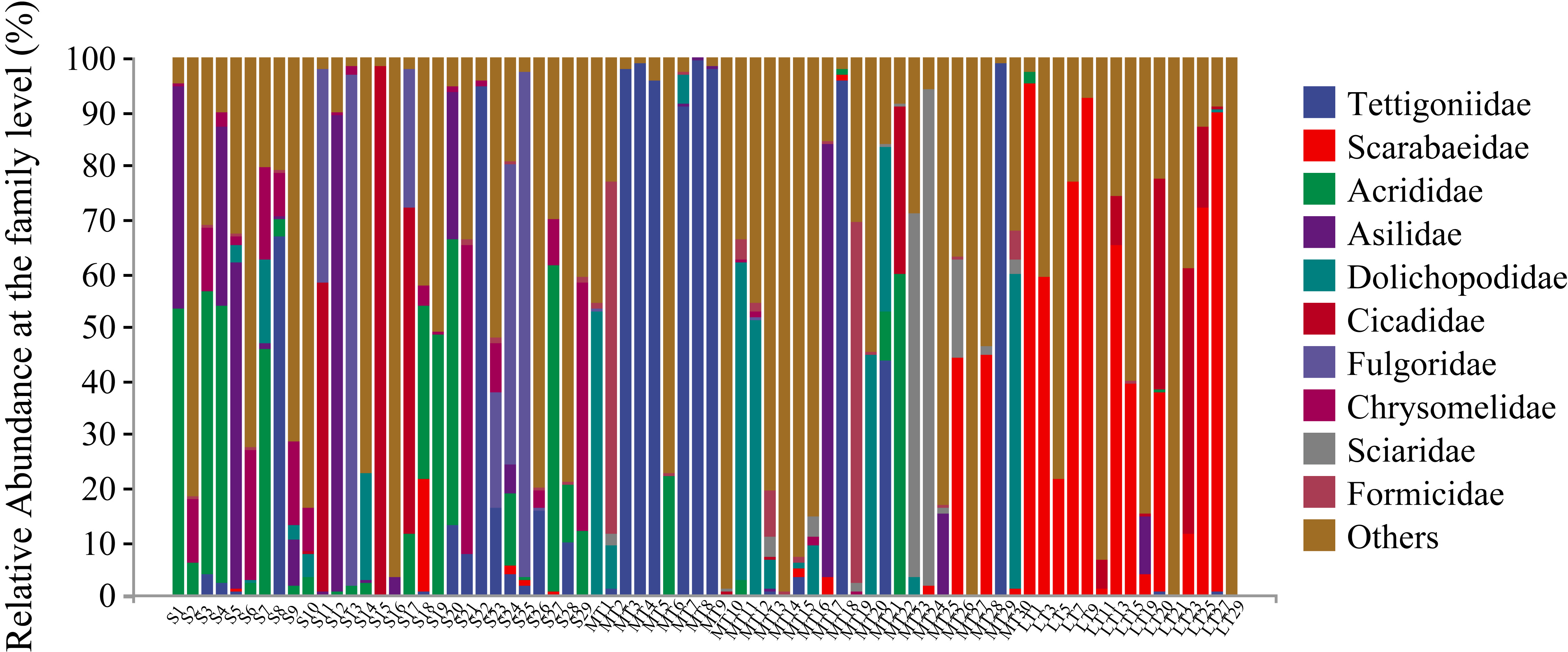

Supplement: Supplementary file 1 — Figure S1 [file ECE3-13-e10031-s009.docx]
