# Supplementary material for: Monitoring insect biodiversity and comparison of sampling strategies using metabarcoding: A case study in the Yanshan Mountains, China
Source: Ecol Evol. 2023 Apr 21;13(4):e10031. doi: 10.1002/ece3.10031 (PMC10121320; doi:10.1002/ece3.10031)
Supplement: Supplementary file 2 — Figure S2 [file ECE3-13-e10031-s018.docx]

**
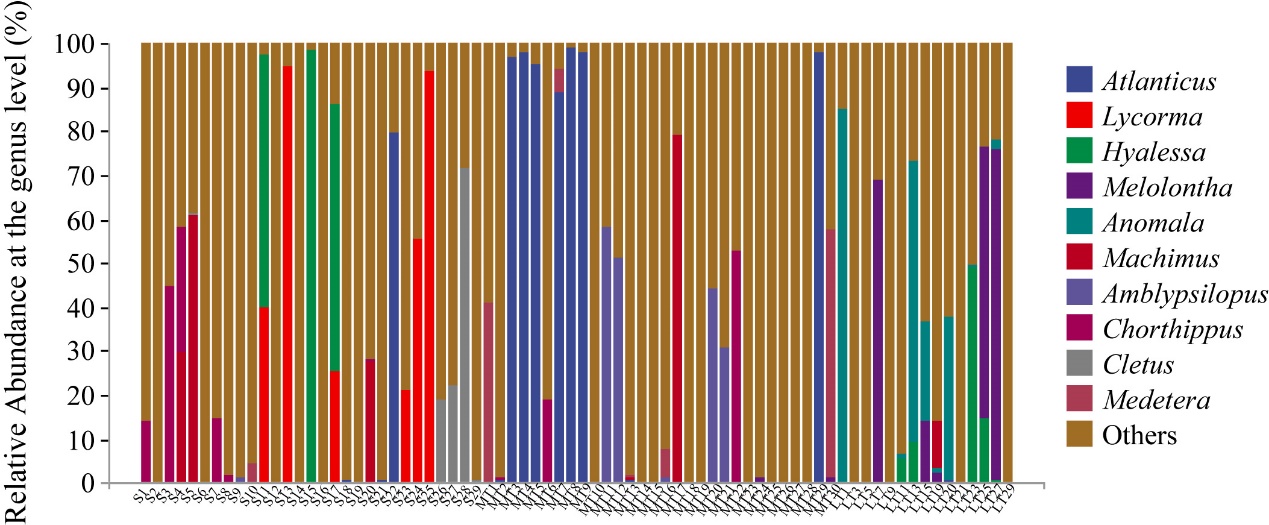
**

(b)


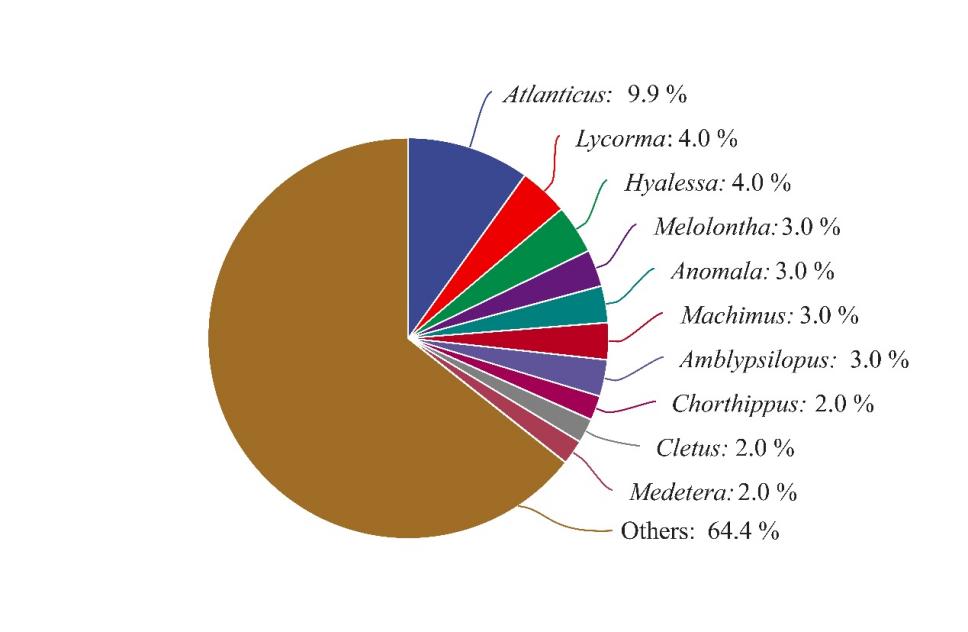


(a)

**FIGURE S2** Statistics of insect community composition at the genus level. (a) Proportion of main genus for Yanshan Mountains was provided in pie chart. (b) The community composition diagram for each sample at the genus level. For the legend on the right, from top to bottom, the abundance of the genus was ranked from most to least.
