# Supplementary material for: Monitoring insect biodiversity and comparison of sampling strategies using metabarcoding: A case study in the Yanshan Mountains, China
Source: Ecol Evol. 2023 Apr 21;13(4):e10031. doi: 10.1002/ece3.10031 (PMC10121320; doi:10.1002/ece3.10031)
Supplement: Supplementary file 3 — Figure S3 [file ECE3-13-e10031-s007.docx]

**
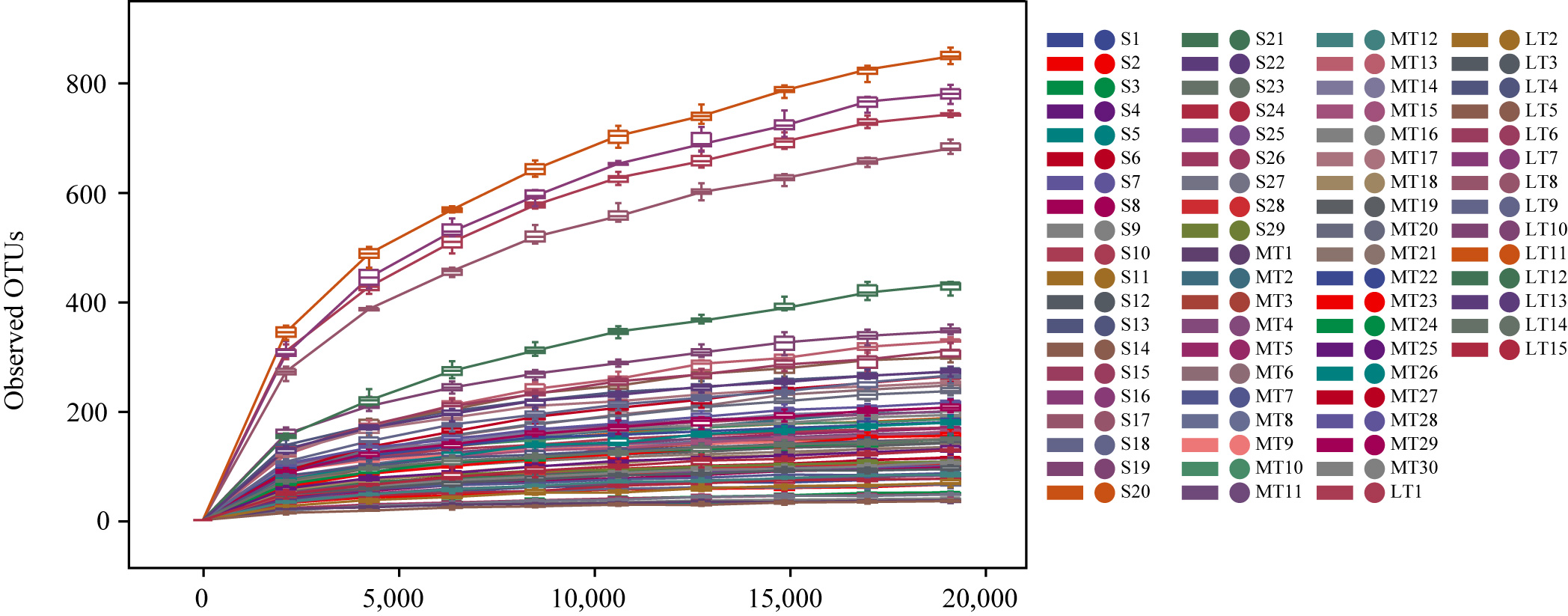
**

**FIGURE S3** Rarefaction curves. Each line in the figure represents a sample. Boxes denote the interquartile range (IQR) between the first and third quartiles (25th and 75th percentiles, respectively) and the line inside denotes the median. S: Sweep netting; MT: Malaise traps; LT: Light traps.
