# Supplementary material for: Monitoring insect biodiversity and comparison of sampling strategies using metabarcoding: A case study in the Yanshan Mountains, China
Source: Ecol Evol. 2023 Apr 21;13(4):e10031. doi: 10.1002/ece3.10031 (PMC10121320; doi:10.1002/ece3.10031)
Supplement: Supplementary file 4 — Figure S4 [file ECE3-13-e10031-s016.docx]

**
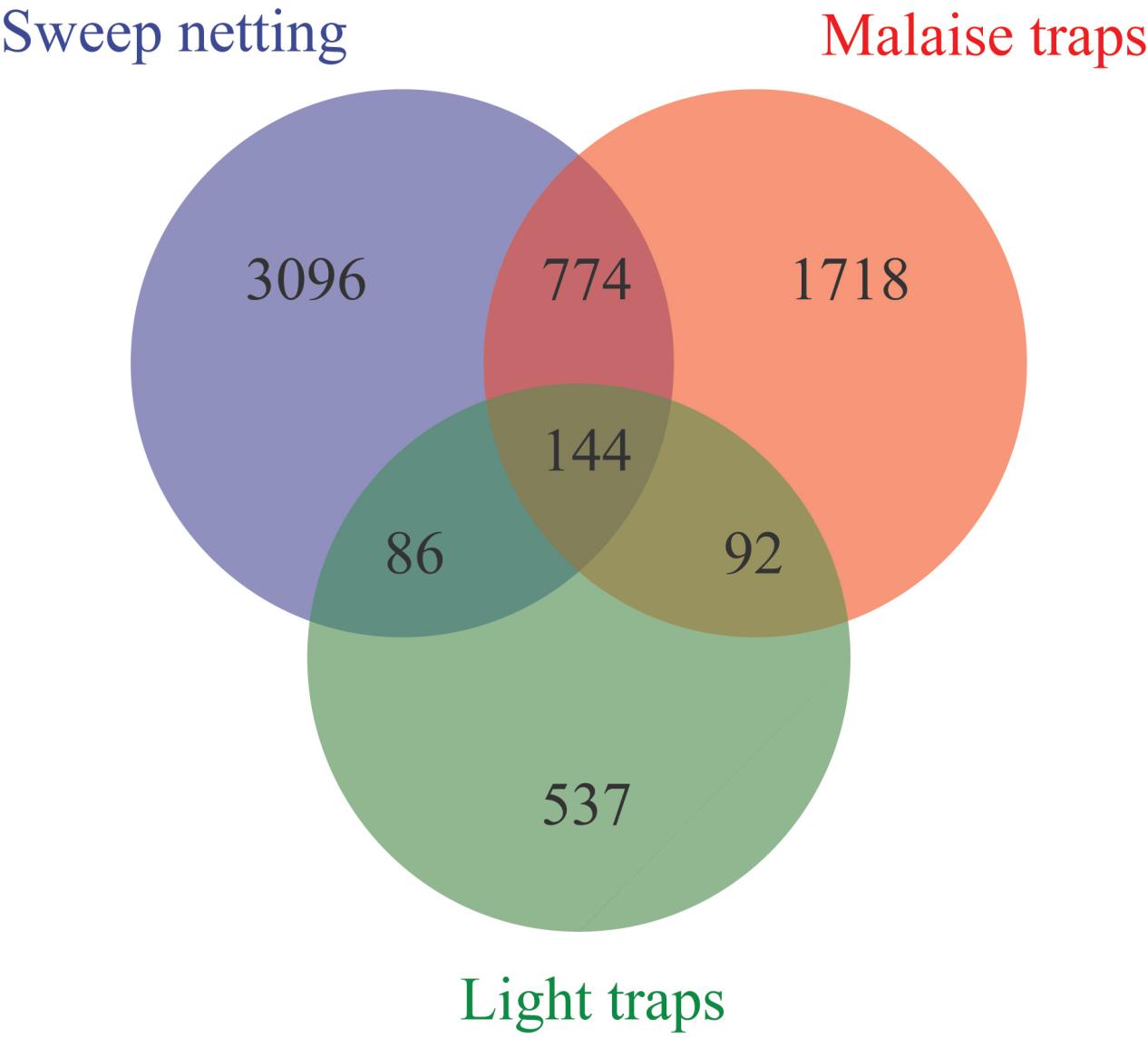
FIGURE S4** Venn diagram of the observed OTUs in the sweep netting group, Malaise trap group and light trap group.
