# Supplementary material for: Monitoring insect biodiversity and comparison of sampling strategies using metabarcoding: A case study in the Yanshan Mountains, China
Source: Ecol Evol. 2023 Apr 21;13(4):e10031. doi: 10.1002/ece3.10031 (PMC10121320; doi:10.1002/ece3.10031)
Supplement: Supplementary file 5 — Figure S5 [file ECE3-13-e10031-s010.docx]

**
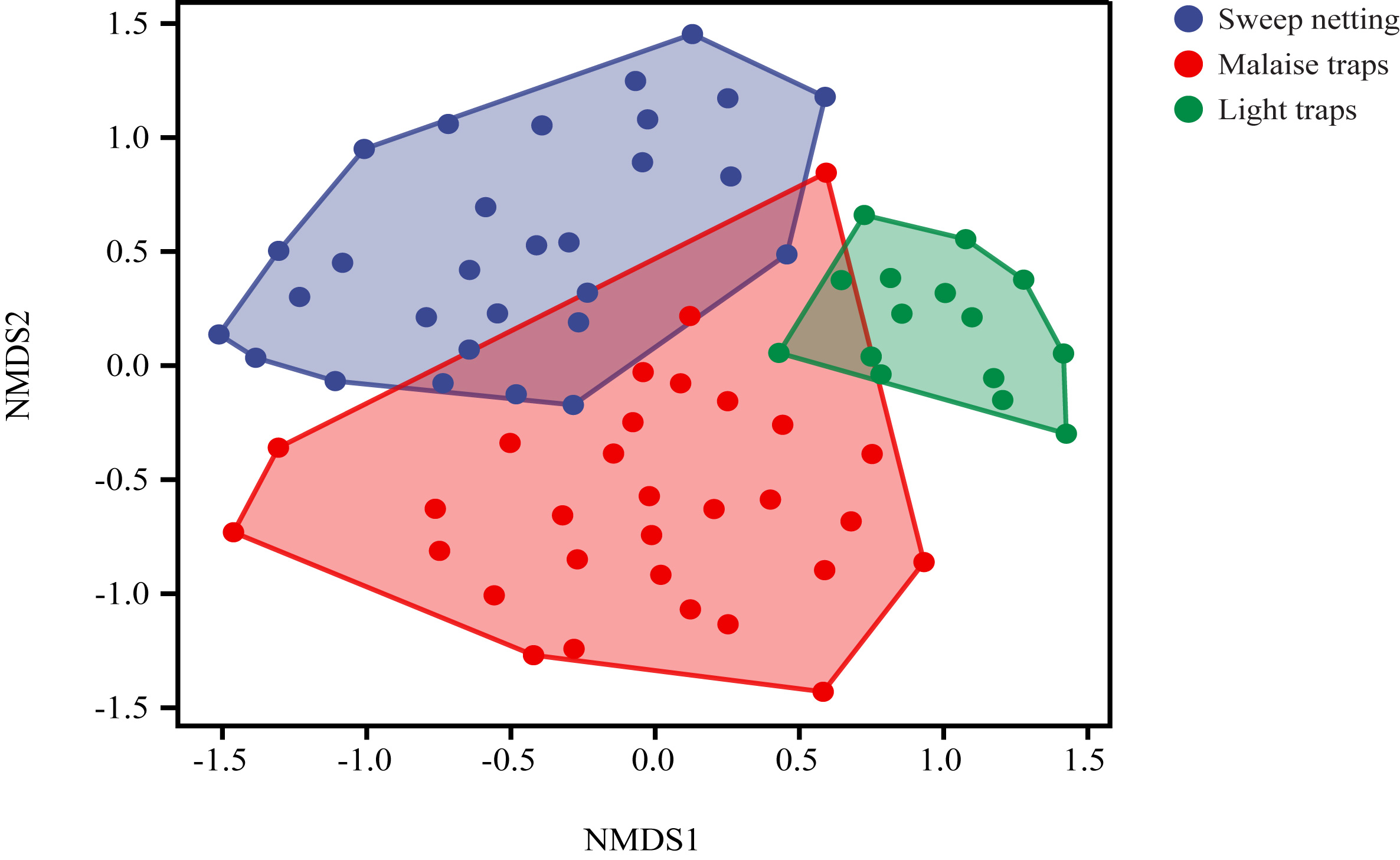
FIGURE S5** Nonmetric multidimensional scaling (NMDS) analysis of insect community similarity recorded from sweep netting, Malaise traps and light traps, based on a Jaccard dissimilarity matrix.
