# Supplementary material for: Monitoring insect biodiversity and comparison of sampling strategies using metabarcoding: A case study in the Yanshan Mountains, China
Source: Ecol Evol. 2023 Apr 21;13(4):e10031. doi: 10.1002/ece3.10031 (PMC10121320; doi:10.1002/ece3.10031)

**FIGURE S6** Heat map displaying the relative abundance of samples comparing three different collection methods. (a): Heat map showing the abundance of 16 orders; (b): Heat map showing the abundance of the top 50 families.


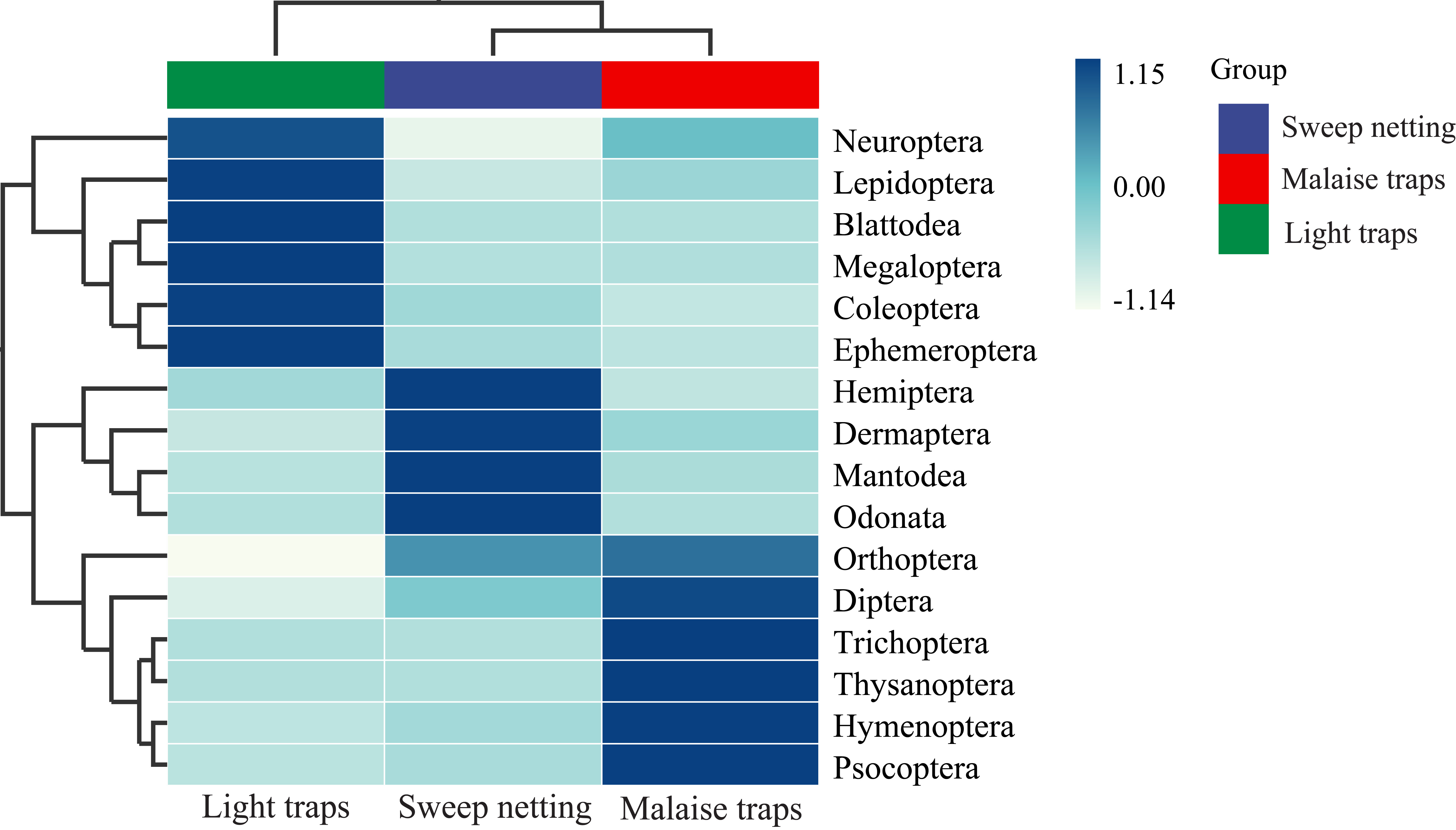


(a)

(b)


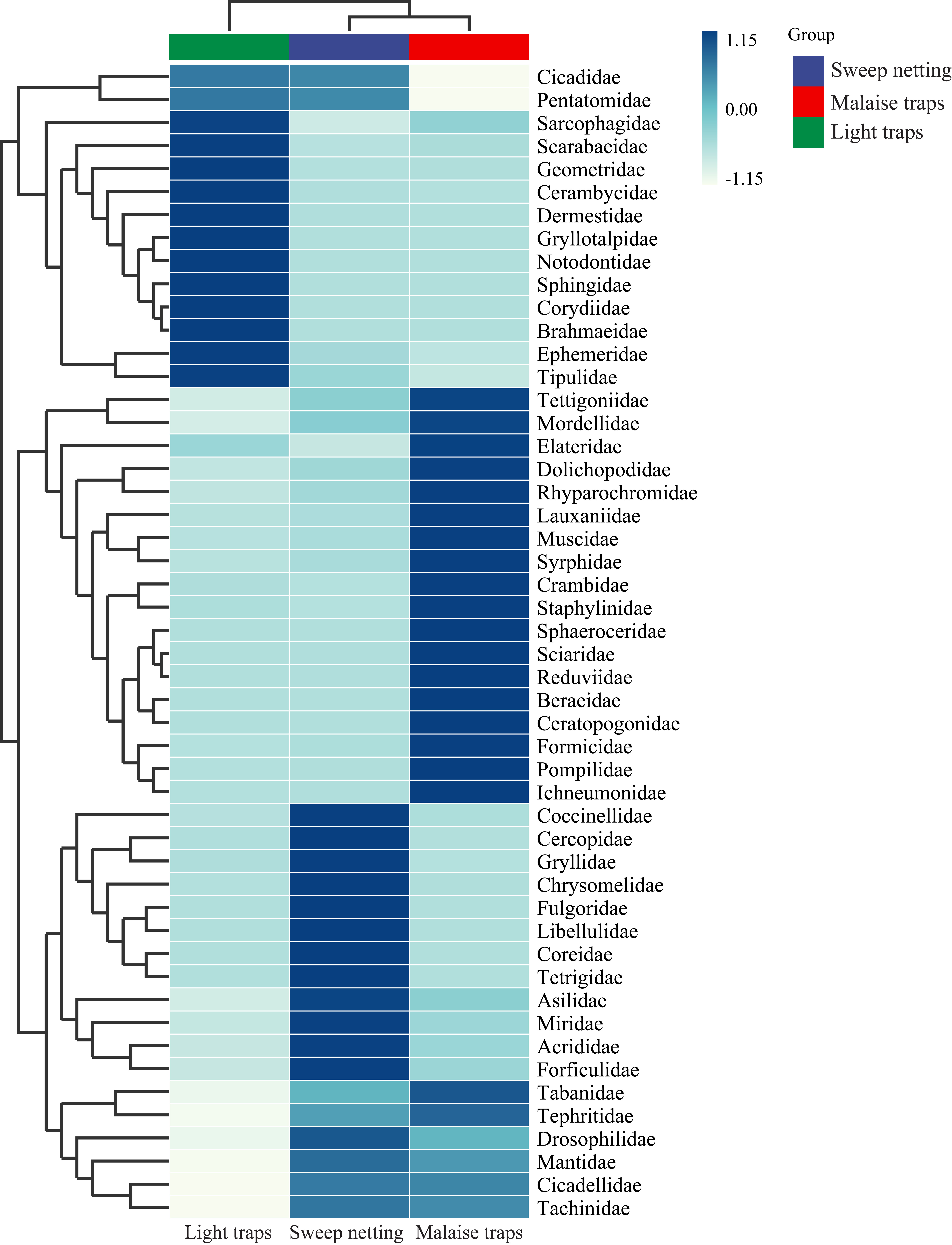

Supplement: Supplementary file 6 — Figure S6 [file ECE3-13-e10031-s002.docx]
