# Supplementary material for: Monitoring insect biodiversity and comparison of sampling strategies using metabarcoding: A case study in the Yanshan Mountains, China
Source: Ecol Evol. 2023 Apr 21;13(4):e10031. doi: 10.1002/ece3.10031 (PMC10121320; doi:10.1002/ece3.10031)
Supplement: Supplementary file 7 — Figure S7 [file ECE3-13-e10031-s008.docx]

**
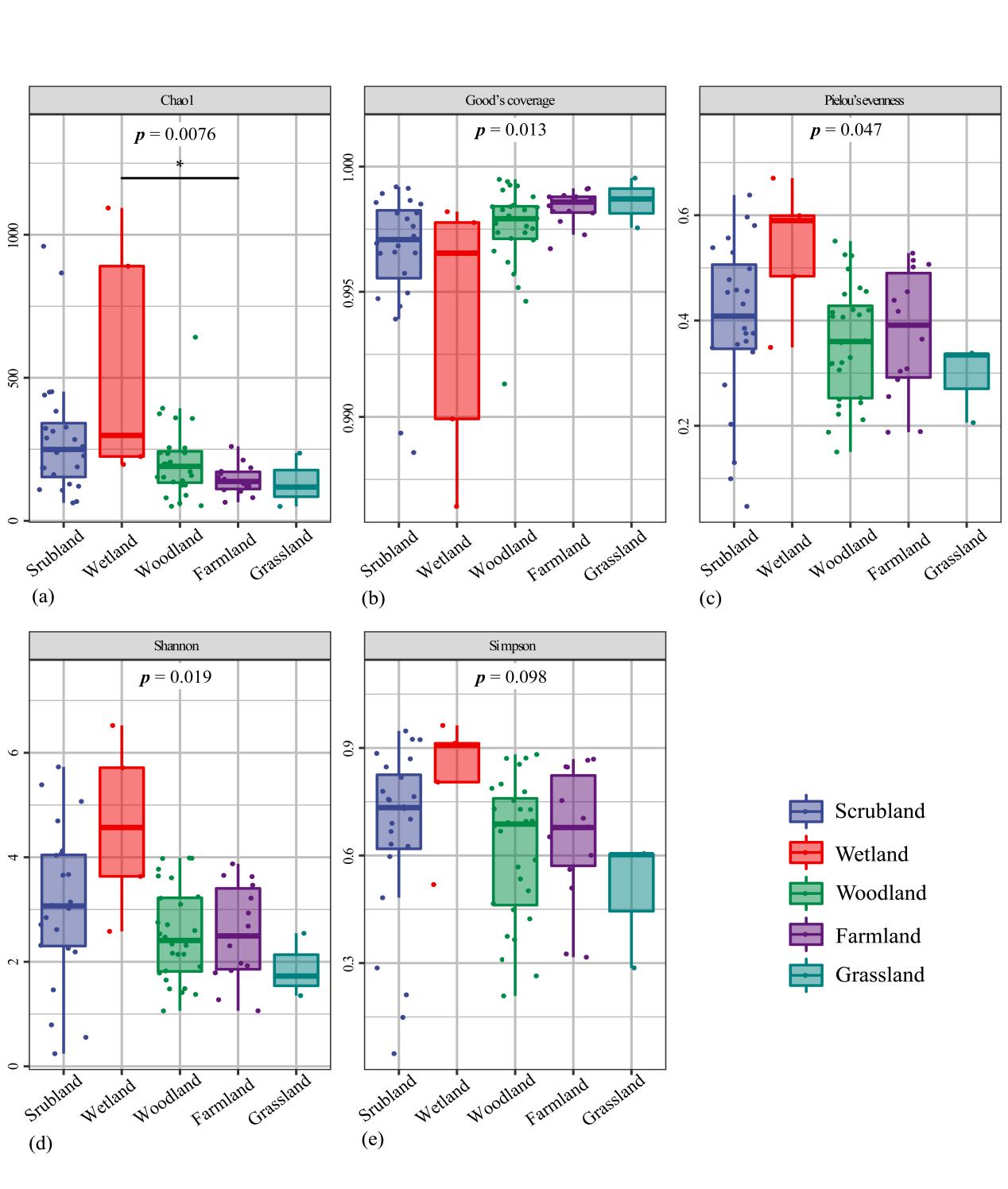
FIGURE S7** Alpha diversity estimates of the five different habitats. The line in the box and the box represents the median and the quartiles, respectively. The number under each diversity index label is the p-value of the Kruskal-Wallis test. The short line represents the significant difference between different collection-method groups calculated using Dunn's test. * represents the degree of difference: * p 〈 0.05, ** p 〈 0.01, *** p 〈 0.001.
