# Supplementary material for: Monitoring insect biodiversity and comparison of sampling strategies using metabarcoding: A case study in the Yanshan Mountains, China
Source: Ecol Evol. 2023 Apr 21;13(4):e10031. doi: 10.1002/ece3.10031 (PMC10121320; doi:10.1002/ece3.10031)
Supplement: Supplementary file 8 — Figure S8 [file ECE3-13-e10031-s015.docx]

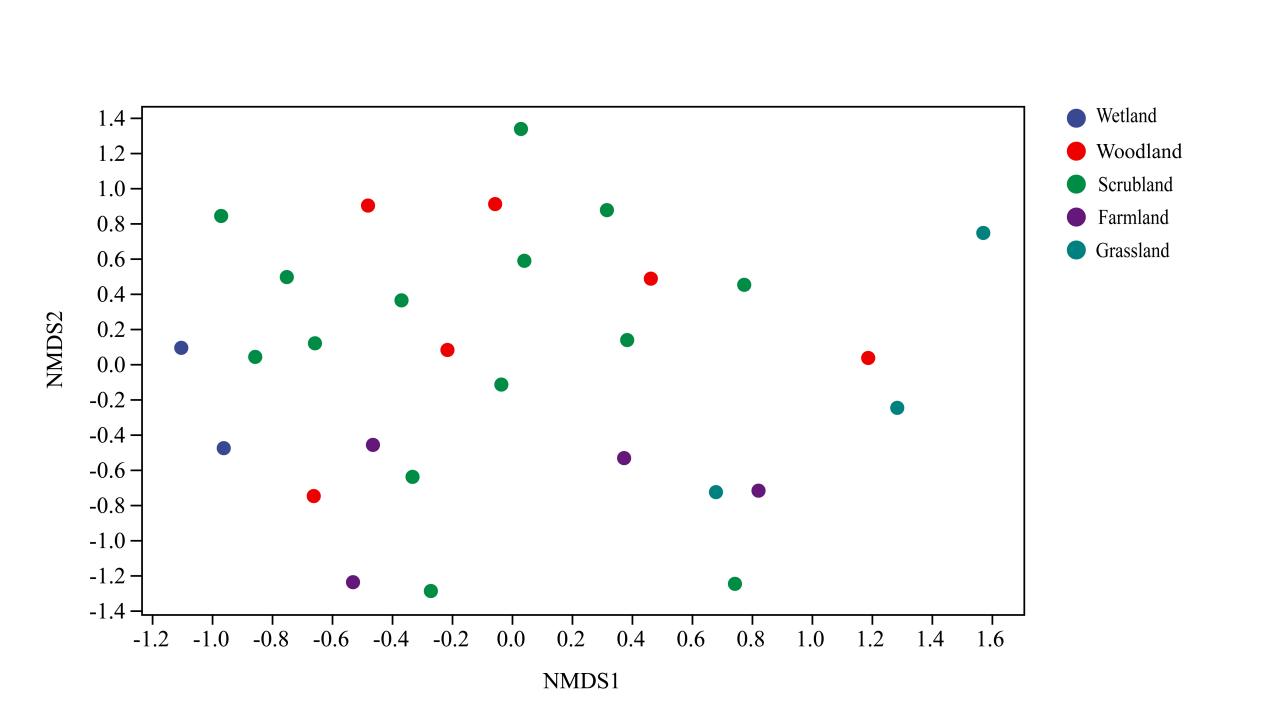


(a)


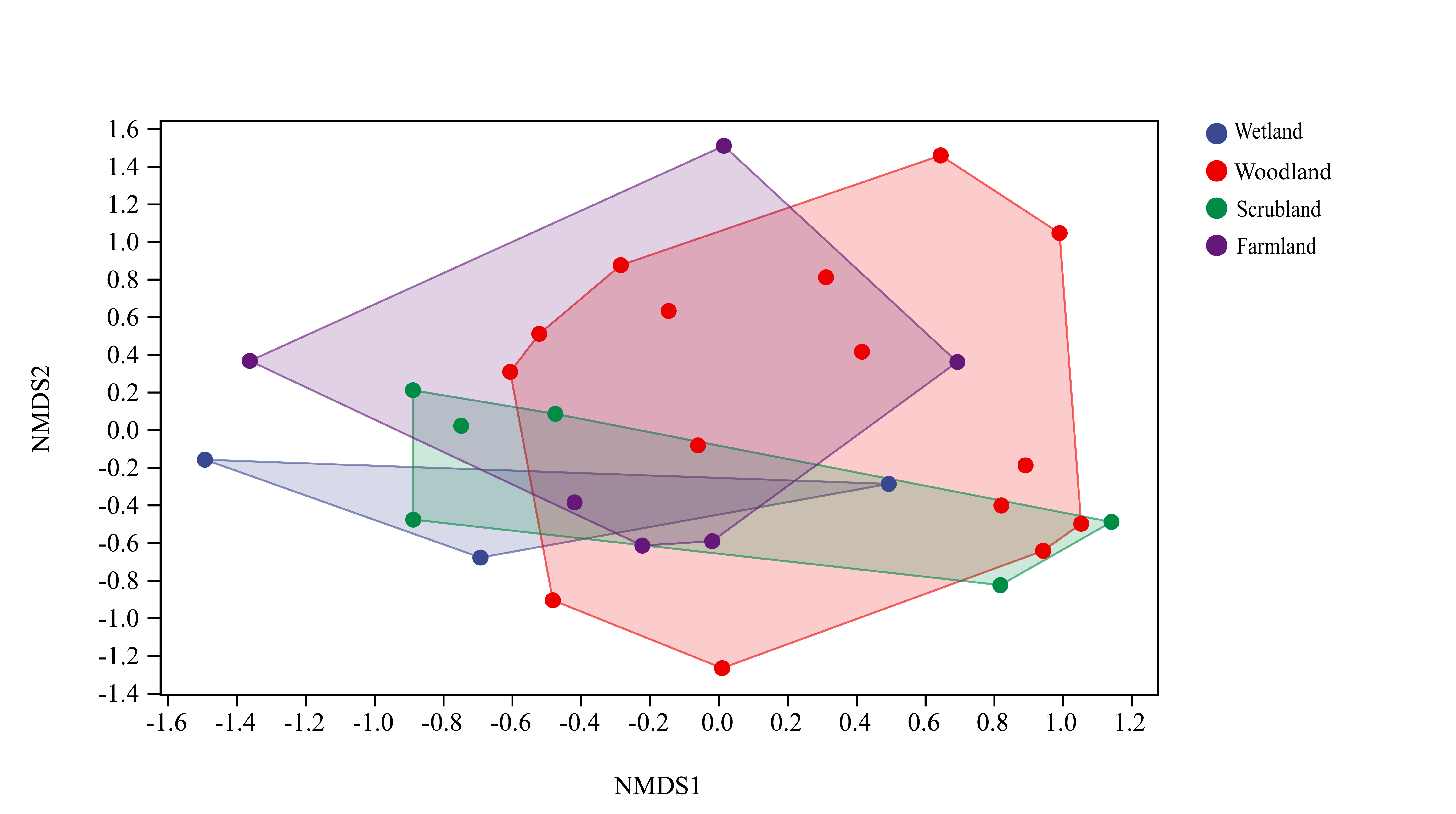


(b)


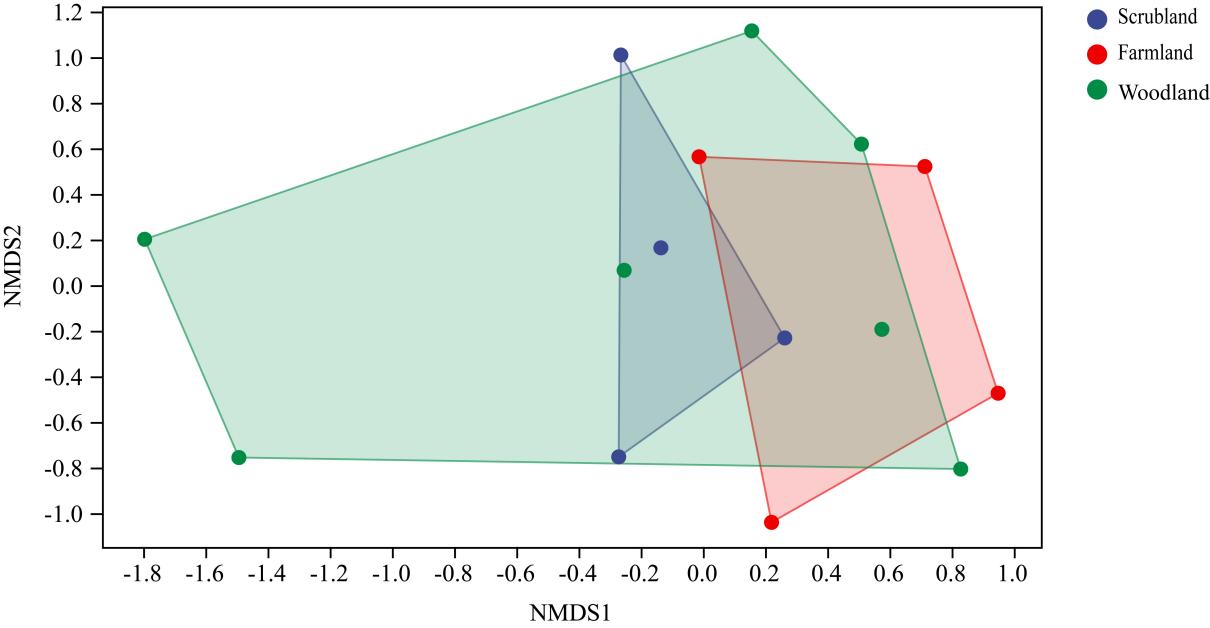


(c)

**FIGURE S8** NMDS analysis of the recovered communities from the different habitats under the same collection method: (a) sweep netting colored by the five habitat categories; (b) Malaise traps colored by the four habitat categories; (c) light traps colored by the three habitat categories; (d) all samples colored by the five habitat categories. Based on Bray–Curtis distance.


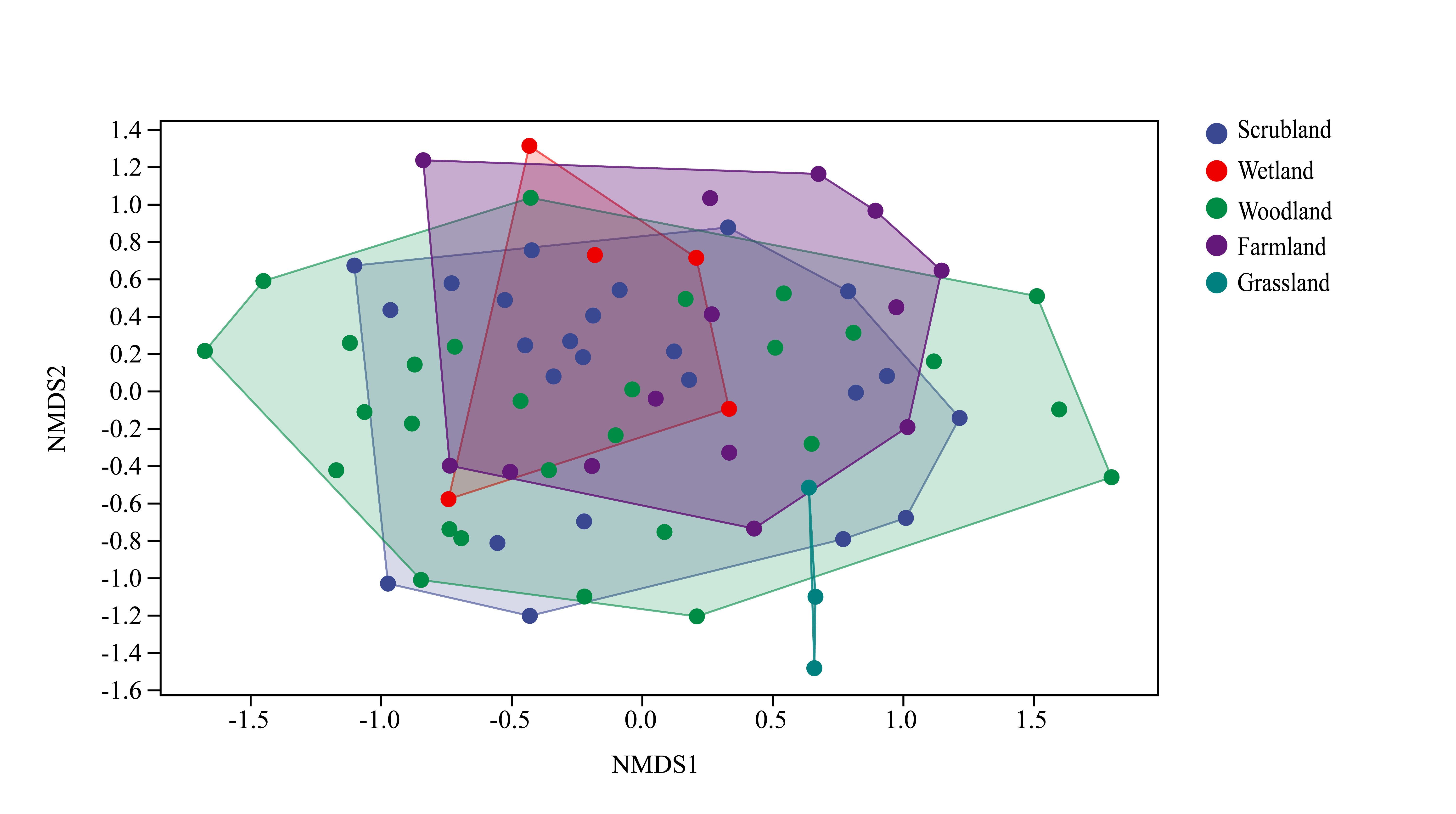


(d)
