# Supplementary material for: Monitoring insect biodiversity and comparison of sampling strategies using metabarcoding: A case study in the Yanshan Mountains, China
Source: Ecol Evol. 2023 Apr 21;13(4):e10031. doi: 10.1002/ece3.10031 (PMC10121320; doi:10.1002/ece3.10031)

(a)

(b)

(c)


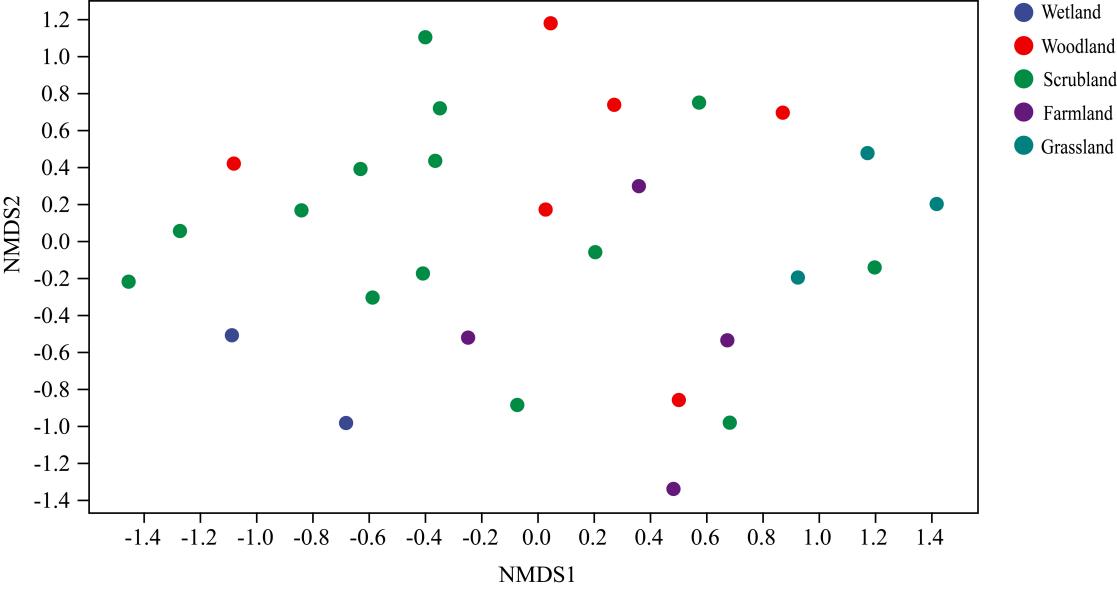

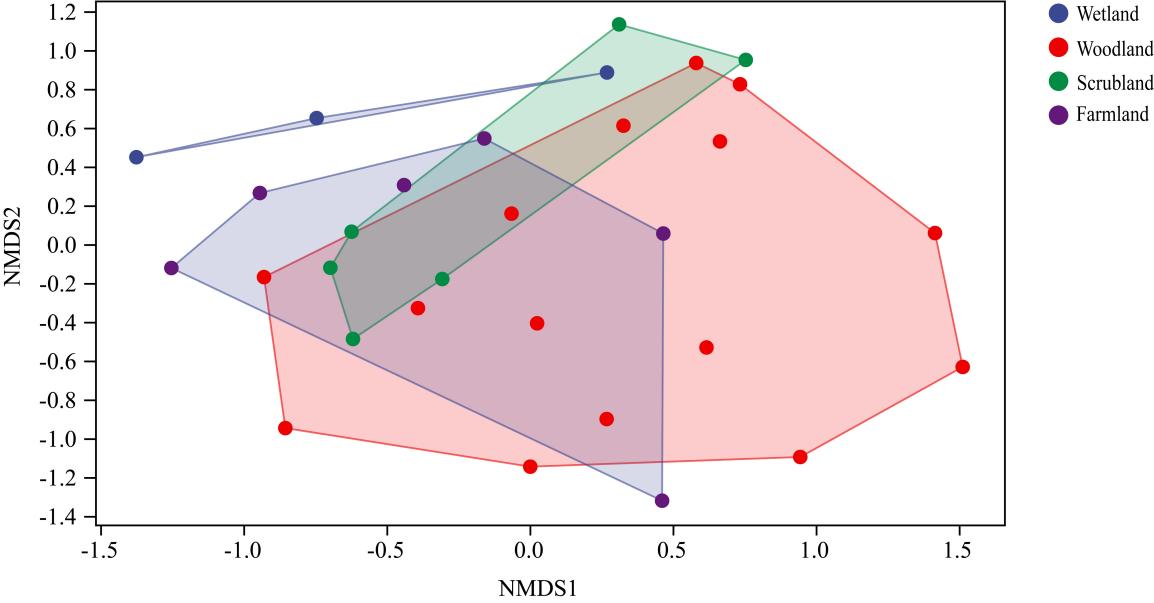

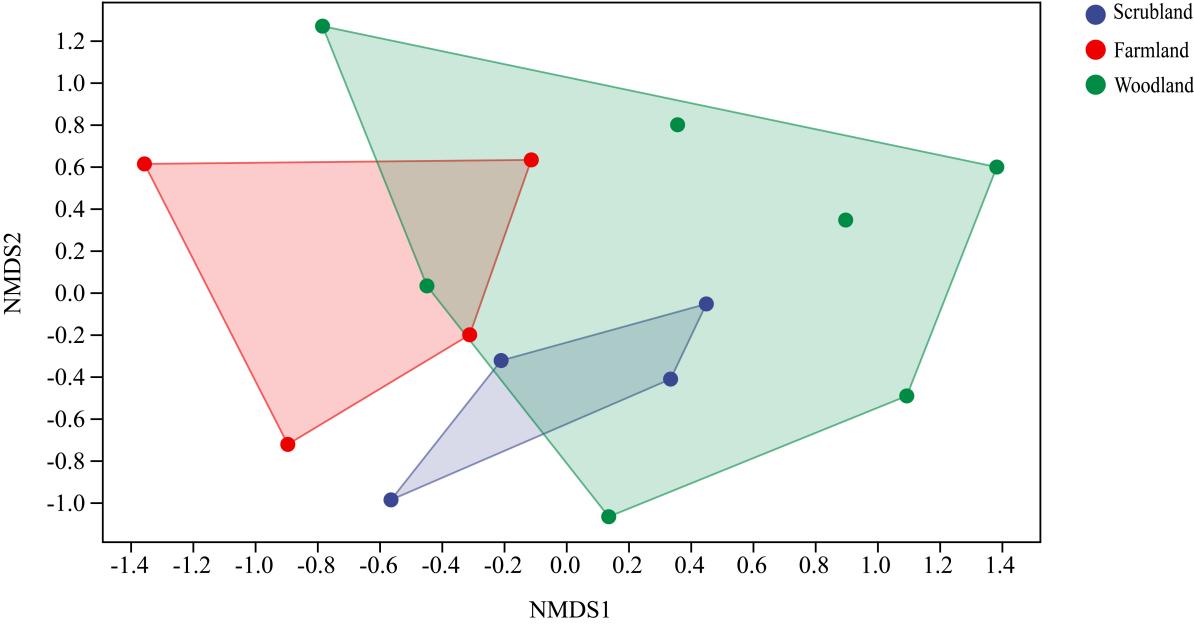


**FIGURE S9** Nonmetric multidimensional scaling (NMDS) analysis of insect community similarity recorded from different habitats, based on a Jaccard dissimilarity matrix: (a) sweep netting colored by the five habitat categories; (b) Malaise traps colored by the four habitat categories; (c) light traps colored by the three habitat categories; (d) all samples colored by the five habitat categories.

(d)


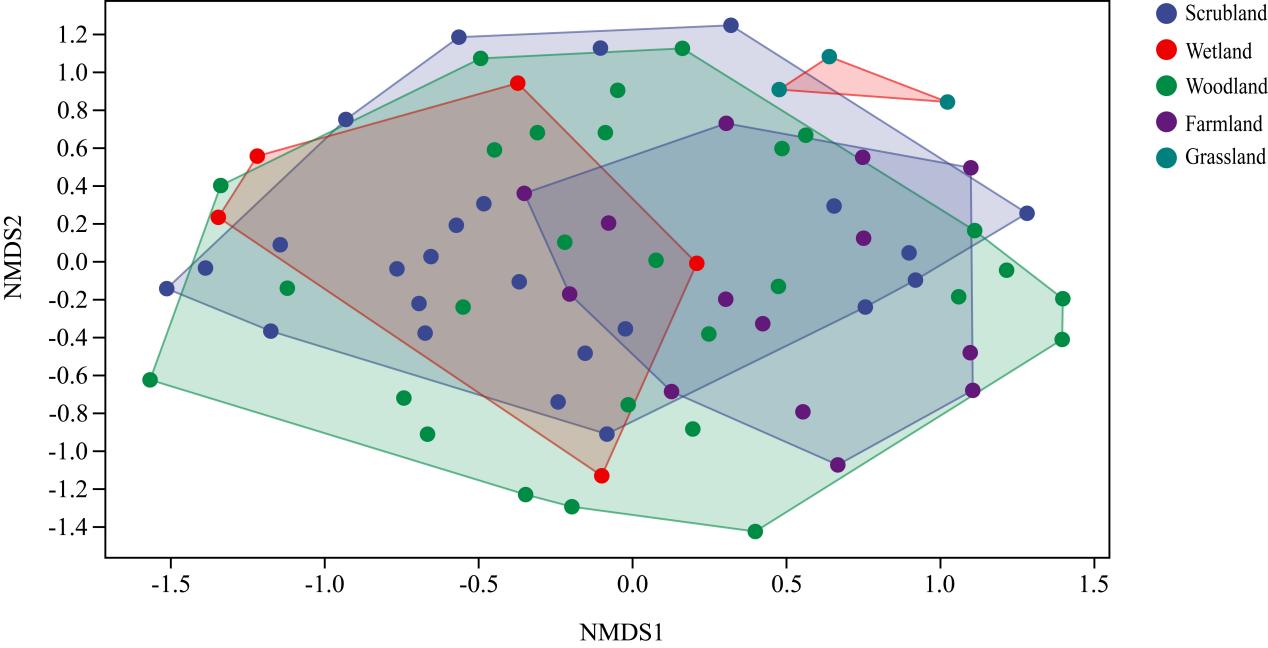

Supplement: Supplementary file 9 — Figure S9 [file ECE3-13-e10031-s013.docx]
