# Supplementary material for: Monitoring insect biodiversity and comparison of sampling strategies using metabarcoding: A case study in the Yanshan Mountains, China
Source: Ecol Evol. 2023 Apr 21;13(4):e10031. doi: 10.1002/ece3.10031 (PMC10121320; doi:10.1002/ece3.10031)
Supplement: Supplementary file 10 — Table S1 [file ECE3-13-e10031-s004.docx]

Table S1 The details of the sampling localities.

| sample ID | location | E | N | ASL (m) | method | Date | habitat |
| --- | --- | --- | --- | --- | --- | --- | --- |
| S1 | Guanting Reservoir National Wetland Park, Hebei | 115.5965 | 40.31924 | 485 | Sweeping method | July 24, 2019 | wetland |
| S2 | Jinjiakou Valley, Huailai County, Hebei | 115.6684 | 40.47699 | 847 | Sweeping method | July 25, 2019 | scrubland |
| S3 | Shijia Village, Chicheng County, Hebei | 115.7379 | 40.60721 | 1080 | Sweeping method | July 28, 2019 | scrubland |
| S4 | Weizigou, Chicheng County, Hebei | 115.9242 | 40.677056 | 1009 | Sweeping method | July 30, 2019 | scrubland |
| S5 | Lichanggou Village, Chicheng County, Hebei | 115.9811 | 40.645859 | 629 | Sweeping method | July 30, 2019 | scrubland |
| S6 | Liergou, Chicheng County, Hebei | 116.1183 | 40.75778 | 736 | Sweeping method | July 31, 2019 | scrubland |
| S7 | Sanjiandi, Chicheng County, Hebei | 116.3712 | 40.45305 | 548 | Sweeping method | July 31, 2019 | wetland |
| S8 | Toudaogou, Chicheng County, Hebei | 116.3522 | 40.97794 | 1102 | Sweeping method | August 2, 2019 | woodland |
| S9 | Diaowo Village, Huairou District, Beijing | 116.5993 | 40.91409 | 520 | Sweeping method | August 3, 2019 | scrubland |
| S10 | Yangshu Valley, Huairou District, Beijing | 116.641 | 40.94741 | 556 | Sweeping method | August 3, 2019 | woodland |
| S11 | Baiquan Mountain, Huairou District, Beijing | 116.6592 | 40.4971 | 256 | Sweeping method | July 26, 2019 | farmland |
| S12 | Shunyi District, Beijing | 116.8769 | 40.1987 | 36 | Sweeping method | July 31, 2019 | scrubland |
| S13 | Chedaoling Village, Miyun District, Beijing | 117.0201 | 40.5947 | 208 | Sweeping method | July 27, 2019 | grassland |
| S14 | Chengezhuang Village, Shunyi District, Beijing | 116.7871 | 40.2399 | 53 | Sweeping method | July 25, 2019 | woodland |
| S15 | Spirit Valley Scenic Spot, Huairou District, Beijing | 116.7954 | 40.5923 | 231 | Sweeping method | July 27, 2019 | scrubland |
| S16 | Xiakou Village, Changping District, Beijing | 116.2423 | 40.3545 | 252 | Sweeping method | July 24, 2019 | grassland |
| S17 | Honglin Village, Huairou District, Beijing | 116.5137 | 40.3296 | 108 | Sweeping method | July 30, 2019 | grassland |
| S18 | Jiuduhe Village, Huairou District, Beijing | 116.3592 | 40.3672 | 182 | Sweeping method | July 26, 2019 | farmland |
| S19 | Qilian Village, Shunyi District, Beijing | 116.8771 | 40.1989 | 33 | Sweeping method | July 31, 2019 | farmland |
| S20 | Yaoqiaoyu Village, Xinchengzi Town, Miyun District, Beijing | 117.3777 | 40.631053 | 435 | Sweeping method | August 4, 2019 | scrubland |
| S21 | Dawafeng Village, Chengde City, Hebei | 117.4251 | 40.616394 | 674 | Sweeping method | August 4, 2019 | scrubland |
| S22 | Yanshi Village, Chengde City, Hebei | 117.497 | 40.499065 | 662 | Sweeping method | July 30, 2019 | woodland |
| S23 | Shijiatai, Luoying Town, Pinggu District, Beijing | 117.2182 | 40.323154 | 487 | Sweeping method | July 27, 2019 | woodland |
| S24 | Xionger village east ditch, Pinggu District, Beijing | 117.147 | 40.27633 | 414 | Sweeping method | July 26, 2019 | scrubland |
| S25 | Baxian Mountain, Ji County, Tianjin | 117.5551 | 40.189067 | 480 | Sweeping method | July 31, 2019 | woodland |
| S26 | Kuliyu Village, Ji County, Tianjin | 117.4433 | 40.17665 | 211 | Sweeping method | August 1, 2019 | scrubland |
| S27 | Guqiangyu Village, Ji County, Tianjin | 117.547 | 40.162984 | 191 | Sweeping method | July 31, 2019 | scrubland |
| S28 | Dongbapinye Village, Chengde City, Hebei | 117.639 | 40.389732 | 670 | Sweeping method | July 30, 2019 | scrubland |
| S29 | Xianjing Village, Zunhua City, Hebei | 117.7342 | 40.18058 | 68 | Sweeping method | July 31, 2019 | farmland |
| MT1 | Guanting Reservoir National Wetland Park, Hebei | 115.6196 | 40.320482 | 473 | Malaise trap | July 24, 2019 | wetland |
| MT2 | Ganquan Village, Huailai County, Hebei | 116.6112 | 40.496943 | 900 | Malaise trap | July 24, 2019 | farmland |
| MT3 | Shijia Village, Chicheng County, Hebei | 115.737 | 40.605606 | 1088 | Malaise trap | July 25, 2019 | scrubland |
| MT4 | Shizikeng, Chicheng County, Hebei | 115.926 | 40.66434 | 930 | Malaise trap | July 26, 2019 | woodland |
| MT5 | Lichanggou Village, Chicheng County, Hebei | 115.9834 | 40.637227 | 749 | Malaise trap | July 26, 2019 | woodland |
| MT6 | Sidaogou Tai, Chicheng County, Hebei | 116.13 | 40.821314 | 937 | Malaise trap | July 27, 2019 | woodland |
| MT7 | Sanjiandi, Chicheng County, Hebei | 116.3615 | 40.7854 | 520 | Malaise trap | July 27, 2019 | wetland |
| MT8 | Xiaogoumen, Chicheng County, Hebei | 116.282 | 40.94719 | 903 | Malaise trap | July 29, 2019 | scrubland |
| MT9 | Beixindian Village, Huairou District, Beijing | 116.5063 | 40.910277 | 805 | Malaise trap | July 29, 2019 | woodland |
| MT10 | Xiaoyangshugou, Huairou district, Beijing | 116.634 | 40.946353 | 548 | Malaise trap | July 29, 2019 | woodland |
| MT11 | Duanshuling Village, Huairou District, Beijing | 116.6599 | 40.4965 | 256 | Malaise trap | August 2, 2019 | scrubland |
| MT12 | Shunyi District, Beijing | 116.7914 | 40.269623 | 65 | Malaise trap | July 31, 2019 | scrubland |
| MT13 | Chedaoling Village, Miyun District, Beijing | 117.0193 | 40.595157 | 208 | Malaise trap | August 2, 2019 | woodland |
| MT14 | Chengezhuang Village, Shunyi District, Beijing | 116.7875 | 40.2394 | 36 | Malaise trap | July 31, 2019 | farmland |
| MT15 | Henglinggen Village, Miyun District, Beijing | 116.7954 | 40.5922 | 219 | Malaise trap | August 2, 2019 | scrubland |
| MT16 | Zhuanghu Village, Bohai Town, Huairou District, Beijing | 116.4698 | 40.4667 | 443 | Malaise trap | July 31, 2019 | woodland |
| MT17 | Xiakou Village, Changping District, Beijing | 116.2316 | 40.346781 | 238 | Malaise trap | July 24, 2019 | woodland |
| MT18 | Calligraphy Villa, Huairou District, Beijing | 116.4719 | 40.294336 | 129 | Malaise trap | July 24, 2019 | wetland |
| MT19 | Jiuduhe Village, Huairou District, Beijing | 116.3589 | 40.3677 | 187 | Malaise trap | July 31, 2019 | farmland |
| MT20 | Shunyi District, Beijing | 116.8765 | 40.1989 | 43 | Malaise trap | July 31, 2019 | woodland |
| MT21 | Yaoqiaoyu Village, Xinchengzi Town, Miyun District, Beijing | 117.3793 | 40.630746 | 437 | Malaise trap | August 4, 2019 | woodland |
| MT22 | Dayanyu Village, Chengde City, Hebei Province | 117.4252 | 40.616376 | 679 | Malaise trap | August 4, 2019 | woodland |
| MT23 | Yanshi Village, Chengde City, Hebei Province | 117.4939 | 40.537742 | 817 | Malaise trap | July 30, 2019 | woodland |
| MT24 | Shijiatai, Luoying Town, Pinggu District, Beijing | 117.2166 | 40.322248 | 535 | Malaise trap | July 27, 2019 | farmland |
| MT25 | Xionger village east ditch, Pinggu District, Beijing | 117.1482 | 40.273541 | 403 | Malaise trap | July 26, 2019 | scrubland |
| MT26 | Baxian Mountain, Ji County, Tianjin | 117.5551 | 40.18905 | 514 | Malaise trap | July 31, 2019 | woodland |
| MT27 | Kuliyu Village, Ji County, Tianjin | 117.4429 | 40.176551 | 232 | Malaise trap | August 1, 2019 | woodland |
| MT28 | Guqiangyu Village, Ji County, Tianjin | 117.5463 | 40.16301 | 177 | Malaise trap | July 31, 2019 | farmland |
| MT29 | Dongbapinye Village, Chengde City, Hebei | 117.6663 | 40.388882 | 604 | Malaise trap | July 29, 2019 | woodland |
| MT30 | Xianjing Village, Zunhua City, Hebei | 117.7547 | 40.183061 | 79 | Malaise trap | July 31, 2019 | farmland |
| LT1 | Chanjiayao Village, Huailai County, Hebei | 115.592 | 40.33192 | 452 | Light traps | July 24, 2019 | farmland |
| LT2 | Shijia Village, Chicheng County, Hebei | 115.7279 | 40.6152 | 1063 | Light traps | July 28, 2019 | farmland |
| LT3 | Lichanggou Village, Chicheng County, Hebei | 115.9829 | 40.647423 | 739 | Light traps | July 30, 2019 | scrubland |
| LT4 | Nanliang, Chicheng County, Hebei | 116.4105 | 40.776282 | 507 | Light traps | August 3, 2019 | farmland |
| LT5 | Sungizi Village, Huairou District, Beijing | 116.5089 | 40.948121 | 751 | Light traps | August 4, 2019 | woodland |
| LT6 | Baiquan Mountain, Huairou District, Beijing | 116.6569 | 40.48846817 | 644 | Light traps | August 8, 2019 | scrubland |
| LT7 | Chedaoling Village, Miyun District, Beijing | 117.0195 | 40.59549713 | 197 | Light traps | August 9, 2019 | woodland |
| LT8 | Duanshuling Village, Huairou District, Beijing | 116.6535 | 40.46109009 | 299 | Light traps | August 11, 2019 | scrubland |
| LT9 | Jiuduhe Village, Huairou District, Beijing | 116.3608 | 40.36573029 | 187 | Light traps | August 7, 2019 | farmland |
| LT10 | Zhumazhuang Village, Shunyi District, Beijing | 116.8785 | 40.18923187 | 45 | Light traps | August 1, 2019 | woodland |
| LT11 | Yaoqiaoyu Village, Xinchengzi Town, Miyun District, Beijing | 117.3765 | 40.633102 | 412 | Light traps | July 27, 2019 | woodland |
| LT12 | Yanshi Village, Chengde City, Hebei | 117.4984 | 40.533752 | 818 | Light traps | August 4, 2019 | woodland |
| LT13 | Xionger village east ditch, Pinggu District, Beijing | 117.1482 | 40.27311 | 403 | Light traps | August 2, 2019 | scrubland |
| LT14 | Kuliyu Village, Ji County, Tianjin | 117.4443 | 40.17701 | 206 | Light traps | July 31, 2019 | woodland |
| LT15 | Dongbapinye Village, Chengde City, Hebei | 117.6621 | 40.388465 | 580 | Light traps | August 2, 2019 | woodland |
| Note: The dates for Malaise traps (MT1-MT30) were the starting dates. | | | | | | | |
