# Supplementary material for: Monitoring insect biodiversity and comparison of sampling strategies using metabarcoding: A case study in the Yanshan Mountains, China
Source: Ecol Evol. 2023 Apr 21;13(4):e10031. doi: 10.1002/ece3.10031 (PMC10121320; doi:10.1002/ece3.10031)
Supplement: Supplementary file 11 — Table S2 [file ECE3-13-e10031-s005.docx]

Table S2 The details for solution after DNA extraction.

| Sample | Subsample | Type | Concentration (ng/μl) | OD260/280 | OD260/230 | Volume (μl) | Total (μg) |
| --- | --- | --- | --- | --- | --- | --- | --- |
| S1 | S1A | DNA | 266.4 | 1.88 | 1.83 | 50 | 13.32 |
|  | S1B | DNA | 231.2 | 1.87 | 1.81 | 50 | 11.56 |
|  | S1C | DNA | 264.9 | 1.87 | 1.58 | 50 | 13.245 |
| S2 | S2A | DNA | 234.7 | 1.92 | 1.83 | 50 | 11.735 |
|  | S2B | DNA | 221.8 | 1.92 | 1.83 | 50 | 11.09 |
|  | S2C | DNA | 210.6 | 1.92 | 1.86 | 50 | 10.53 |
| S3 | S3A | DNA | 211 | 1.87 | 1.76 | 50 | 10.55 |
|  | S3B | DNA | 200.9 | 1.87 | 1.75 | 50 | 10.045 |
|  | S3C | DNA | 223.5 | 1.88 | 1.67 | 50 | 11.175 |
| S4 | S4A | DNA | 218.4 | 1.88 | 1.77 | 50 | 10.92 |
|  | S4B | DNA | 232.5 | 1.87 | 1.64 | 50 | 11.625 |
|  | S4C | DNA | 223 | 1.89 | 1.89 | 50 | 11.15 |
| S5 | S5A | DNA | 155.9 | 1.87 | 1.67 | 50 | 7.795 |
|  | S5B | DNA | 128.3 | 1.88 | 1.58 | 50 | 6.415 |
|  | S5C | DNA | 135.8 | 1.87 | 1.63 | 50 | 6.79 |
| S6 | S6A | DNA | 138.1 | 1.88 | 1.62 | 50 | 6.905 |
|  | S6B | DNA | 132.7 | 1.87 | 1.59 | 50 | 6.635 |
|  | S6C | DNA | 118.7 | 1.87 | 1.59 | 50 | 5.935 |
| S7 | S7A | DNA | 218.1 | 1.88 | 1.87 | 50 | 10.905 |
|  | S7B | DNA | 225.5 | 1.87 | 1.89 | 50 | 11.275 |
|  | S7C | DNA | 213.9 | 1.88 | 1.85 | 50 | 10.695 |
| S8 | S8A | DNA | 195.7 | 1.86 | 1.83 | 50 | 9.785 |
|  | S8B | DNA | 190 | 1.87 | 1.82 | 50 | 9.5 |
|  | S8C | DNA | 187 | 1.87 | 1.83 | 50 | 9.35 |
| S9 | S9A | DNA | 141.3 | 1.87 | 1.49 | 50 | 7.065 |
|  | S9B | DNA | 155.3 | 1.87 | 1.74 | 50 | 7.765 |
|  | S9C | DNA | 176.5 | 1.87 | 1.75 | 50 | 8.825 |
| S10 | S10A | DNA | 174.7 | 1.88 | 1.76 | 50 | 8.735 |
|  | S10B | DNA | 131.7 | 1.87 | 1.57 | 50 | 6.585 |
|  | S10C | DNA | 158.5 | 1.89 | 1.78 | 50 | 7.925 |
| S11 | S11A | DNA | 36.8 | 1.99 | 0.89 | 50 | 1.84 |
|  | S11B | DNA | 45 | 1.96 | 0.99 | 50 | 2.25 |
|  | S11C | DNA | 49.5 | 1.96 | 1.08 | 50 | 2.475 |
| S12 | S12A | DNA | 56.3 | 1.91 | 1.11 | 50 | 2.815 |
|  | S12B | DNA | 49.4 | 1.91 | 1.04 | 50 | 2.47 |
|  | S12C | DNA | 87 | 1.88 | 1.4 | 50 | 4.35 |
| S13 | S13A | DNA | 77.7 | 1.93 | 1.22 | 50 | 3.885 |
|  | S13B | DNA | 56.2 | 1.92 | 1.05 | 50 | 2.81 |
|  | S13C | DNA | 47.3 | 1.92 | 0.99 | 50 | 2.365 |
| S14 | S14A | DNA | 36.6 | 1.91 | 0.82 | 50 | 1.83 |
|  | S14B | DNA | 47.9 | 1.9 | 1.01 | 50 | 2.395 |
|  | S14C | DNA | 50.6 | 1.92 | 0.99 | 50 | 2.53 |
| S15 | S15A | DNA | 41.9 | 1.91 | 0.95 | 50 | 2.095 |
|  | S15B | DNA | 32.7 | 1.94 | 0.81 | 50 | 1.635 |
|  | S15C | DNA | 22.8 | 1.92 | 0.62 | 50 | 1.14 |
| S16 | S16A | DNA | 34 | 1.94 | 0.8 | 50 | 1.7 |
|  | S16B | DNA | 20.3 | 2 | 0.54 | 50 | 1.015 |
|  | S16C | DNA | 32.4 | 1.87 | 0.78 | 50 | 1.62 |
| S17 | S18A | DNA | 39.8 | 1.91 | 0.83 | 50 | 1.99 |
|  | S18B | DNA | 47.2 | 1.93 | 1 | 50 | 2.36 |
|  | S18C | DNA | 59.2 | 1.91 | 1.11 | 50 | 2.96 |
| S18 | S19A | DNA | 24.3 | 1.9 | 0.6 | 50 | 1.215 |
|  | S19B | DNA | 23.7 | 1.95 | 0.62 | 50 | 1.185 |
|  | S19C | DNA | 12.2 | 2.03 | 0.41 | 50 | 0.61 |
| S19 | S20A | DNA | 21.2 | 1.95 | 0.61 | 50 | 1.06 |
|  | S20B | DNA | 29.2 | 1.86 | 0.63 | 50 | 1.46 |
|  | S20C | DNA | 21.5 | 2.03 | 0.61 | 50 | 1.075 |
| S20 | S21A | DNA | 141.4 | 1.86 | 1.66 | 50 | 7.07 |
|  | S21B | DNA | 164.3 | 1.88 | 1.74 | 50 | 8.215 |
|  | S21C | DNA | 163.8 | 1.88 | 1.71 | 50 | 8.19 |
| S21 | S22A | DNA | 101.7 | 1.89 | 1.51 | 50 | 5.085 |
|  | S22B | DNA | 92.4 | 1.87 | 1.48 | 50 | 4.62 |
|  | S22C | DNA | 99.4 | 1.89 | 1.47 | 50 | 4.97 |
| S22 | S23A | DNA | 102.3 | 1.88 | 1.53 | 50 | 5.115 |
|  | S23B | DNA | 137.7 | 1.86 | 1.25 | 50 | 6.885 |
|  | S23C | DNA | 135.5 | 1.88 | 1.6 | 50 | 6.775 |
| S23 | S24A | DNA | 62.4 | 1.89 | 1.26 | 50 | 3.12 |
|  | S24B | DNA | 78.3 | 1.89 | 1.39 | 50 | 3.915 |
|  | S24C | DNA | 63.1 | 1.9 | 1.24 | 50 | 3.155 |
| S24 | S25A | DNA | 174.6 | 1.87 | 1.82 | 50 | 8.73 |
|  | S25B | DNA | 128.6 | 1.89 | 1.67 | 50 | 6.43 |
|  | S25C | DNA | 146.6 | 1.88 | 1.71 | 50 | 7.33 |
| S25 | S26A | DNA | 123.4 | 1.88 | 1.63 | 50 | 6.17 |
|  | S26B | DNA | 111.2 | 1.89 | 1.57 | 50 | 5.56 |
|  | S26C | DNA | 124.6 | 1.89 | 1.64 | 50 | 6.23 |
| S26 | S27A | DNA | 127 | 1.9 | 1.65 | 50 | 6.35 |
|  | S27B | DNA | 109.4 | 1.87 | 1.52 | 50 | 5.47 |
|  | S27C | DNA | 108.9 | 1.9 | 1.65 | 50 | 5.445 |
| S27 | S28A | DNA | 117.6 | 1.89 | 1.69 | 50 | 5.88 |
|  | S28B | DNA | 109.3 | 1.89 | 1.59 | 50 | 5.465 |
|  | S28C | DNA | 120.4 | 1.88 | 1.62 | 50 | 6.02 |
| S28 | S29A | DNA | 171.8 | 1.88 | 1.83 | 50 | 8.59 |
|  | S29B | DNA | 150 | 1.87 | 1.75 | 50 | 7.5 |
|  | S29C | DNA | 145.3 | 1.86 | 1.71 | 50 | 7.265 |
| S29 | S30A | DNA | 77.5 | 1.88 | 1.22 | 50 | 3.875 |
|  | S30B | DNA | 94.8 | 1.87 | 1.36 | 50 | 4.74 |
|  | S30C | DNA | 114.6 | 1.86 | 1.48 | 50 | 5.73 |
| MT1 | MT1A | DNA | 24.1 | 1.94 | 0.58 | 50 | 1.205 |
|  | MT1B | DNA | 33.7 | 1.9 | 1.02 | 50 | 1.685 |
|  | MT1C | DNA | 38.1 | 1.86 | 0.81 | 50 | 1.905 |
| MT2 | MT2A | DNA | 7 | 1.85 | 0.2 | 50 | 0.35 |
|  | MT2B | DNA | 3.8 | 2.14 | 0.11 | 50 | 0.19 |
|  | MT2C | DNA | 4.2 | 2.59 | 0.09 | 50 | 0.21 |
| MT3 | MT3A | DNA | 118.6 | 1.86 | 1.5 | 50 | 5.93 |
|  | MT3B | DNA | 132.4 | 1.85 | 1.57 | 50 | 6.62 |
|  | MT3C | DNA | 156.3 | 1.87 | 1.71 | 50 | 7.815 |
| MT4 | MT4A | DNA | 134 | 1.87 | 1.68 | 50 | 6.7 |
|  | MT4B | DNA | 128.9 | 1.88 | 1.63 | 50 | 6.445 |
|  | MT4C | DNA | 146.5 | 1.87 | 1.59 | 50 | 7.325 |
| MT5 | MT5A | DNA | 138.9 | 1.86 | 1.63 | 50 | 6.945 |
|  | MT5B | DNA | 143.5 | 1.85 | 1.64 | 50 | 7.175 |
|  | MT5C | DNA | 118.7 | 1.86 | 1.56 | 50 | 5.935 |
| MT6 | MT6A | DNA | 135.3 | 1.85 | 1.64 | 50 | 6.765 |
|  | MT6B | DNA | 151.8 | 1.89 | 1.81 | 50 | 7.59 |
|  | MT6C | DNA | 151.6 | 1.88 | 1.71 | 50 | 7.58 |
| MT7 | MT7A | DNA | 152.4 | 1.87 | 1.71 | 50 | 7.62 |
|  | MT7B | DNA | 139.9 | 1.87 | 1.66 | 50 | 6.995 |
|  | MT7C | DNA | 176.8 | 1.87 | 1.72 | 50 | 8.84 |
| MT8 | MT8A | DNA | 132.5 | 1.87 | 1.66 | 50 | 6.625 |
|  | MT8B | DNA | 155.9 | 1.87 | 1.77 | 50 | 7.795 |
|  | MT8C | DNA | 149.3 | 1.86 | 1.74 | 50 | 7.465 |
| MT9 | MT9A | DNA | 143.5 | 1.86 | 1.71 | 50 | 7.175 |
|  | MT9B | DNA | 147.8 | 1.86 | 1.73 | 50 | 7.39 |
|  | MT9C | DNA | 149.8 | 1.86 | 1.73 | 50 | 7.49 |
| MT10 | MT10A | DNA | 69.8 | 1.88 | 1.3 | 50 | 3.49 |
|  | MT10B | DNA | 54.9 | 1.9 | 1.21 | 50 | 2.745 |
|  | MT10C | DNA | 79.6 | 1.88 | 1.41 | 50 | 3.98 |
| MT11 | MT11A | DNA | 64.5 | 1.91 | 1.28 | 50 | 3.225 |
|  | MT11B | DNA | 64.4 | 1.89 | 1.11 | 50 | 3.22 |
|  | MT11C | DNA | 81.5 | 1.89 | 1.29 | 50 | 4.075 |
| MT12 | MT12A | DNA | 21.8 | 2 | 0.66 | 50 | 1.09 |
|  | MT12B | DNA | 28.3 | 1.94 | 0.83 | 50 | 1.415 |
|  | MT12C | DNA | 30.2 | 1.91 | 0.85 | 50 | 1.51 |
| MT13 | MT13A | DNA | 12.5 | 2.03 | 0.43 | 50 | 0.625 |
|  | MT13B | DNA | 11.1 | 1.98 | 0.41 | 50 | 0.555 |
|  | MT13C | DNA | 11.5 | 2.12 | 0.39 | 50 | 0.575 |
| MT14 | MT14A | DNA | 21.1 | 1.96 | 0.41 | 50 | 1.055 |
|  | MT14B | DNA | 19.2 | 1.95 | 0.62 | 50 | 0.96 |
|  | MT14C | DNA | 20.3 | 2.04 | 0.66 | 50 | 1.015 |
| MT15 | MT15A | DNA | 43.2 | 1.92 | 0.98 | 50 | 2.16 |
|  | MT15B | DNA | 39.4 | 1.95 | 0.83 | 50 | 1.97 |
|  | MT15C | DNA | 31.1 | 1.92 | 0.79 | 50 | 1.555 |
| MT16 | MT16A | DNA | 41.3 | 1.94 | 1.01 | 50 | 2.065 |
|  | MT16B | DNA | 53 | 1.92 | 1.17 | 50 | 2.65 |
|  | MT16C | DNA | 43.8 | 1.92 | 1.04 | 50 | 2.19 |
| MT17 | MT17A | DNA | 48.6 | 1.92 | 1.09 | 50 | 2.43 |
|  | MT17B | DNA | 49.4 | 1.96 | 0.63 | 50 | 2.47 |
|  | MT17C | DNA | 81.3 | 1.9 | 1.37 | 50 | 4.065 |
| MT18 | MT18A | DNA | 25.8 | 1.95 | 0.8 | 50 | 1.29 |
|  | MT18B | DNA | 32.8 | 1.91 | 0.9 | 50 | 1.64 |
|  | MT18C | DNA | 43.5 | 1.9 | 1.06 | 50 | 2.175 |
| MT19 | MT19A | DNA | 16.9 | 2.02 | 0.52 | 50 | 0.845 |
|  | MT19B | DNA | 17.7 | 1.99 | 0.51 | 50 | 0.885 |
|  | MT19C | DNA | 20.5 | 1.98 | 0.56 | 50 | 1.025 |
| MT20 | MT20A | DNA | 24.4 | 1.92 | 0.68 | 50 | 1.22 |
|  | MT20B | DNA | 18.9 | 1.97 | 0.57 | 50 | 0.945 |
|  | MT20C | DNA | 25.2 | 2 | 0.78 | 50 | 1.26 |
| MT21 | MT21A | DNA | 162.3 | 1.88 | 1.72 | 50 | 8.115 |
|  | MT21B | DNA | 160.6 | 1.88 | 1.66 | 50 | 8.03 |
|  | MT21C | DNA | 167.3 | 1.88 | 1.86 | 50 | 8.365 |
| MT22 | MT22A | DNA | 147.2 | 1.88 | 1.84 | 50 | 7.36 |
|  | MT22B | DNA | 136.7 | 1.87 | 1.81 | 50 | 6.835 |
|  | MT22C | DNA | 153.3 | 1.87 | 1.83 | 50 | 7.665 |
| MT23 | MT23A | DNA | 90.3 | 1.89 | 1.38 | 50 | 4.515 |
|  | MT23B | DNA | 106.1 | 1.89 | 1.47 | 50 | 5.305 |
|  | MT23C | DNA | 115.4 | 1.89 | 1.55 | 50 | 5.77 |
| MT24 | MT24A | DNA | 5.6 | 2.04 | 0.21 | 50 | 0.28 |
|  | MT24B | DNA | 3.2 | 2.06 | 0.11 | 50 | 0.16 |
|  | MT24C | DNA | 2.9 | 2.13 | 0.07 | 50 | 0.145 |
| MT25 | MT25A | DNA | 22.2 | 2.03 | 0.71 | 50 | 1.11 |
|  | MT25B | DNA | 44.1 | 1.94 | 1.11 | 50 | 2.205 |
|  | MT25C | DNA | 21.1 | 1.99 | 0.73 | 50 | 1.055 |
| MT26 | MT26A | DNA | 53.4 | 1.97 | 1.28 | 50 | 2.67 |
|  | MT26B | DNA | 60.3 | 1.97 | 1.32 | 50 | 3.015 |
|  | MT26C | DNA | 34.9 | 1.85 | 0.76 | 50 | 1.745 |
| MT27 | MT27A | DNA | 142.6 | 1.87 | 1.78 | 50 | 7.13 |
|  | MT27B | DNA | 171 | 1.88 | 1.81 | 50 | 8.55 |
|  | MT27C | DNA | 171.9 | 1.88 | 1.79 | 50 | 8.595 |
| MT28 | MT28A | DNA | 53.7 | 1.9 | 1.18 | 50 | 2.685 |
|  | MT28B | DNA | 50 | 1.9 | 1.14 | 50 | 2.5 |
|  | MT28C | DNA | 58.6 | 1.91 | 1.26 | 50 | 2.93 |
| MT29 | MT29A | DNA | 156.7 | 1.86 | 1.77 | 50 | 7.835 |
|  | MT29B | DNA | 133.5 | 1.86 | 1.7 | 50 | 6.675 |
|  | MT29C | DNA | 169 | 1.87 | 1.84 | 50 | 8.45 |
| MT30 | MT30A | DNA | 2.5 | 1.98 | 0.06 | 50 | 0.125 |
|  | MT30B | DNA | 2.2 | 2.01 | 0.09 | 50 | 0.11 |
|  | MT30C | DNA | 2.3 | 2.07 | 0.05 | 50 | 0.115 |
| LT1 | LT1A | DNA | 72.8 | 1.94 | 1.3 | 50 | 3.64 |
|  | LT1B | DNA | 110.7 | 1.91 | 1.55 | 50 | 5.535 |
|  | LT1C | DNA | 111.4 | 1.9 | 1.57 | 50 | 5.57 |
| LT2 | LT2A | DNA | 56 | 1.93 | 1.16 | 50 | 2.8 |
|  | LT2B | DNA | 41.4 | 1.92 | 0.94 | 50 | 2.07 |
|  | LT2C | DNA | 51.4 | 1.91 | 1.1 | 50 | 2.57 |
| LT3 | LT3A | DNA | 51 | 1.92 | 1.08 | 50 | 2.55 |
|  | LT3B | DNA | 30.3 | 1.92 | 0.79 | 50 | 1.515 |
|  | LT3C | DNA | 38.4 | 1.92 | 0.93 | 50 | 1.92 |
| LT4 | LT4A | DNA | 94.4 | 1.92 | 1.53 | 50 | 4.72 |
|  | LT4B | DNA | 88.1 | 1.9 | 1.47 | 50 | 4.405 |
|  | LT4C | DNA | 111.7 | 1.88 | 1.58 | 50 | 5.585 |
| LT5 | LT5A | DNA | 38.1 | 1.94 | 0.91 | 50 | 1.905 |
|  | LT5B | DNA | 66.9 | 1.86 | 0.86 | 50 | 3.345 |
|  | LT5C | DNA | 55.6 | 1.88 | 0.78 | 50 | 2.78 |
| LT6 | LT6A | DNA | 69.3 | 1.86 | 0.94 | 50 | 3.465 |
|  | LT6B | DNA | 75.6 | 1.87 | 0.98 | 50 | 3.78 |
|  | LT6C | DNA | 65.5 | 1.86 | 0.91 | 50 | 3.275 |
| LT7 | LT7A | DNA | 43.7 | 1.9 | 0.5 | 50 | 2.185 |
|  | LT7B | DNA | 40.4 | 1.9 | 0.59 | 50 | 2.02 |
|  | LT7C | DNA | 34.7 | 1.9 | 0.58 | 50 | 1.735 |
| LT8 | LT8A | DNA | 37.9 | 1.91 | 0.62 | 50 | 1.895 |
|  | LT8B | DNA | 31.7 | 1.89 | 0.58 | 50 | 1.585 |
|  | LT8C | DNA | 47.8 | 1.74 | 0.71 | 50 | 2.39 |
| LT9 | LT9A | DNA | 41.4 | 1.91 | 0.68 | 50 | 2.07 |
|  | LT9B | DNA | 44.6 | 1.88 | 0.72 | 50 | 2.23 |
|  | LT9C | DNA | 55.1 | 1.86 | 0.86 | 50 | 2.755 |
| LT10 | LT10A | DNA | 34.7 | 1.9 | 0.54 | 50 | 1.735 |
|  | LT10B | DNA | 26.8 | 1.9 | 0.41 | 50 | 1.34 |
|  | LT10C | DNA | 40.5 | 1.86 | 0.7 | 50 | 2.025 |
| LT11 | LT11A | DNA | 9 | 1.98 | 0.13 | 50 | 0.45 |
|  | LT11B | DNA | 19.8 | 1.92 | 0.53 | 50 | 0.99 |
|  | LT11C | DNA | 10.1 | 1.8 | 0.18 | 50 | 0.505 |
| LT12 | LT12A | DNA | 57.1 | 1.92 | 0.8 | 50 | 2.855 |
|  | LT12B | DNA | 60.9 | 1.94 | 0.99 | 50 | 3.045 |
|  | LT12C | DNA | 68.6 | 1.91 | 0.99 | 50 | 3.43 |
| LT13 | LT13A | DNA | 44.1 | 1.92 | 0.71 | 50 | 2.205 |
|  | LT13B | DNA | 67.9 | 1.88 | 0.92 | 50 | 3.395 |
|  | LT13C | DNA | 47.6 | 1.9 | 0.79 | 50 | 2.38 |
| LT14 | LT14A | DNA | 73.2 | 1.87 | 1.05 | 50 | 3.66 |
|  | LT14B | DNA | 83 | 1.88 | 0.94 | 50 | 4.15 |
|  | LT14C | DNA | 61.2 | 1.88 | 0.93 | 50 | 3.06 |
| LT15 | LT15A | DNA | 78.1 | 1.9 | 1.08 | 50 | 3.905 |
|  | LT15B | DNA | 66.3 | 1.9 | 0.99 | 50 | 3.315 |
|  | LT15C | DNA | 84.5 | 1.87 | 1.12 | 50 | 4.225 |
