# Supplementary material for: Monitoring insect biodiversity and comparison of sampling strategies using metabarcoding: A case study in the Yanshan Mountains, China
Source: Ecol Evol. 2023 Apr 21;13(4):e10031. doi: 10.1002/ece3.10031 (PMC10121320; doi:10.1002/ece3.10031)
Supplement: Supplementary file 12 — Table S3 [file ECE3-13-e10031-s017.docx]

**Table S5.** Characteristics of 74 samples after sequencing and OTUs picking.

| **Sample ID** | **Input** | **HQS** | **Nonsingleton** | **Rarefication** | **OTUs** | **Insecta OTUs** |
| --- | --- | --- | --- | --- | --- | --- |
| S1 | 56,628 | 32,422 | 32,290 | 19,117 | 845 | 832 |
| S2 | 58,002 | 55,795 | 55,712 | 19,117 | 449 | 436 |
| S3 | 56,484 | 48,444 | 48,220 | 19,117 | 964 | 956 |
| S4 | 58,181 | 54,281 | 54,087 | 19,117 | 871 | 864 |
| S5 | 56,379 | 54,232 | 54,170 | 19,117 | 394 | 370 |
| S6 | 52,860 | 51,569 | 51,495 | 19,117 | 455 | 440 |
| S7 | 55,045 | 51,642 | 51,461 | 19,117 | 1090 | 1079 |
| S8 | 54,989 | 53,243 | 53,164 | 19,117 | 626 | 610 |
| S9 | 58,055 | 50,116 | 50,053 | 19,117 | 349 | 331 |
| S10 | 52,613 | 50,917 | 50,894 | 19,117 | 194 | 185 |
| S11 | 52,032 | 50,006 | 49,981 | 19,117 | 194 | 187 |
| S12 | 62,985 | 60,602 | 60,586 | 19,117 | 110 | 106 |
| S13 | 60,268 | 57,595 | 57,570 | 19,117 | 128 | 126 |
| S14 | 56,815 | 55,004 | 54,988 | 19,117 | 188 | 178 |
| S15 | 54,958 | 54,035 | 54,027 | 19,117 | 65 | 53 |
| S16 | 53,713 | 52,879 | 52,875 | 19,117 | 49 | 47 |
| S17 | 39,358 | 34,252 | 34,220 | 19,117 | 234 | 216 |
| S18 | 37,904 | 34,500 | 34,464 | 19,117 | 192 | 175 |
| S19 | 37,115 | 36,409 | 36,396 | 19,117 | 101 | 99 |
| S20 | 54,528 | 52,991 | 52,939 | 19,117 | 452 | 449 |
| S21 | 59,542 | 57,383 | 57,351 | 19,117 | 293 | 284 |
| S22 | 53,273 | 50,492 | 50,468 | 19,117 | 209 | 205 |
| S23 | 56,789 | 55,477 | 55,442 | 19,117 | 254 | 240 |
| S24 | 54,499 | 53,261 | 53,231 | 19,117 | 324 | 319 |
| S25 | 55,946 | 54,318 | 54,301 | 19,117 | 197 | 195 |
| S26 | 48,201 | 43,801 | 43,772 | 19,117 | 180 | 175 |
| S27 | 55,737 | 49,021 | 48,996 | 19,117 | 260 | 257 |
| S28 | 56,426 | 55,205 | 55,155 | 19,117 | 332 | 328 |
| S29 | 52,876 | 51,600 | 51,569 | 19,117 | 183 | 171 |
| MT1 | 51,274 | 48,590 | 48,557 | 19,117 | 253 | 219 |
| MT2 | 53,475 | 51,848 | 51,832 | 19,117 | 240 | 226 |
| MT3 | 57,672 | 55,395 | 55,359 | 19,117 | 190 | 189 |
| MT4 | 53,966 | 51,665 | 51,640 | 19,117 | 180 | 177 |
| MT5 | 54,839 | 51,908 | 51,868 | 19,117 | 266 | 259 |
| MT6 | 58,944 | 57,121 | 57,088 | 19,117 | 365 | 359 |
| MT7 | 51,752 | 45,096 | 45,006 | 19,117 | 275 | 268 |
| MT8 | 49,293 | 41,994 | 41,921 | 19,117 | 260 | 259 |
| MT9 | 58,429 | 55,510 | 55,477 | 19,117 | 231 | 225 |
| MT10 | 56,425 | 54,493 | 54,460 | 19,117 | 146 | 134 |
| MT11 | 57,674 | 55,964 | 55,917 | 19,117 | 274 | 242 |
| MT12 | 61,853 | 59,492 | 59,456 | 19,117 | 251 | 235 |
| MT13 | 54,317 | 52,757 | 52,721 | 19,117 | 381 | 353 |
| MT14 | 55,777 | 54,606 | 54,585 | 19,117 | 120 | 108 |
| MT15 | 52,270 | 48,630 | 48,591 | 19,117 | 199 | 193 |
| MT16 | 51,965 | 50,712 | 50,685 | 19,117 | 199 | 183 |
| MT17 | 52,176 | 50,113 | 50,095 | 19,117 | 100 | 94 |
| MT18 | 49,657 | 45,208 | 45,168 | 19,117 | 207 | 199 |
| MT19 | 63,960 | 60,464 | 60,436 | 19,117 | 154 | 128 |
| MT20 | 51,132 | 49,684 | 49,664 | 19,117 | 119 | 112 |
| MT21 | 48,581 | 45,281 | 45,221 | 19,117 | 348 | 336 |
| MT22 | 46,644 | 44,196 | 44,156 | 19,117 | 369 | 365 |
| MT23 | 50,917 | 47,389 | 47,310 | 19,117 | 274 | 246 |
| MT24 | 46,604 | 45,626 | 45,616 | 19,117 | 120 | 107 |
| MT25 | 47,678 | 46,553 | 46,528 | 19,117 | 161 | 143 |
| MT26 | 57,422 | 55,353 | 55,319 | 19,117 | 101 | 81 |
| MT27 | 38,212 | 37,424 | 37,398 | 19,117 | 140 | 116 |
| MT28 | 36,280 | 35,578 | 35,559 | 19,117 | 83 | 74 |
| MT29 | 29,366 | 24,775 | 24,700 | 19,117 | 208 | 201 |
| MT30 | 30,415 | 27,354 | 27,324 | 19,117 | 122 | 104 |
| LT1 | 31,221 | 29,560 | 29,505 | 19,117 | 129 | 126 |
| LT2 | 29,060 | 26,228 | 26,173 | 19,117 | 158 | 154 |
| LT3 | 62,938 | 61,321 | 61,273 | 19,117 | 110 | 107 |
| LT4 | 28,329 | 25,737 | 25,703 | 19,117 | 117 | 114 |
| LT5 | 67,402 | 64,924 | 64,888 | 19,117 | 70 | 68 |
| LT6 | 29,573 | 27,426 | 27,398 | 19,117 | 56 | 53 |
| LT7 | 65,508 | 63,335 | 63,289 | 19,117 | 164 | 157 |
| LT8 | 28,951 | 27,436 | 27,389 | 19,117 | 117 | 115 |
| LT9 | 31,747 | 29,028 | 29,011 | 19,117 | 61 | 60 |
| LT10 | 33,483 | 31,828 | 31,778 | 19,117 | 140 | 134 |
| LT11 | 30,276 | 27,410 | 27,382 | 19,117 | 42 | 40 |
| LT12 | 29,927 | 28,292 | 28,233 | 19,117 | 133 | 125 |
| LT13 | 30,916 | 28,549 | 28,510 | 19,117 | 112 | 111 |
| LT14 | 66,145 | 62,406 | 62,365 | 19,117 | 146 | 139 |
| LT15 | 28,464 | 26,204 | 26,161 | 19,117 | 45 | 44 |
| Tatol | 3,685,110 | 3,471,955 | 3,468,592 | 1,414,658 | 7,427 | 7,083 |
| Mean number | 49,798.78 | 46,918.31 | 46,872.86 | 19,117 | 244.84 | 235.07 |

**Input:** The number of raw sequences obtained from MiSeqPE300. **HQS**: High-quality sequences that have been merged, quality filtering and chimera detection. **Nonsingleton:** High-quality sequences after singleton removal. **Rarefication:** The number of High-quality sequences after rarefied all samples to equal depth. **OTUs:** OTUs identified at 97% similarity thresholds. **Insect OTUs:** Only OTUs from Insecta.
