# Supplementary material for: Monitoring insect biodiversity and comparison of sampling strategies using metabarcoding: A case study in the Yanshan Mountains, China
Source: Ecol Evol. 2023 Apr 21;13(4):e10031. doi: 10.1002/ece3.10031 (PMC10121320; doi:10.1002/ece3.10031)
Supplement: Supplementary file 15 — Table S6 [file ECE3-13-e10031-s014.docx]

**Table S6.** Alpha diversity indexes for each sample.

| **Sample ID** | **Chao1** | **Good’s coverage** | **Pielou's evenness** | **Shannon** | **Simpson** |
| --- | --- | --- | --- | --- | --- |
| S1 | 890.184 | 0.98992 | 0.599263 | 5.71354 | 0.907452 |
| S2 | 439.604 | 0.994419 | 0.498425 | 4.12293 | 0.818014 |
| S3 | 959.828 | 0.988576 | 0.596501 | 5.73003 | 0.923181 |
| S4 | 866.536 | 0.989355 | 0.538482 | 5.06911 | 0.869558 |
| S5 | 383.238 | 0.994952 | 0.375568 | 3.02224 | 0.631599 |
| S6 | 450.055 | 0.994727 | 0.6383 | 5.38742 | 0.947348 |
| S7 | 1093.47 | 0.986426 | 0.670439 | 6.52317 | 0.962733 |
| S8 | 641.904 | 0.991317 | 0.455237 | 3.98276 | 0.854489 |
| S9 | 327.573 | 0.996537 | 0.580475 | 4.69647 | 0.924198 |
| S10 | 191.14 | 0.997934 | 0.523105 | 3.77268 | 0.871033 |
| S11 | 212.484 | 0.99728 | 0.255617 | 1.78962 | 0.561408 |
| S12 | 109.089 | 0.998567 | 0.130057 | 0.793786 | 0.211573 |
| S13 | 117.982 | 0.998708 | 0.206104 | 1.35476 | 0.28678 |
| S14 | 172.338 | 0.998457 | 0.550918 | 3.97713 | 0.881881 |
| S15 | 62.8324 | 0.999194 | 0.046951 | 0.243016 | 0.047555 |
| S16 | 50.5833 | 0.99954 | 0.334484 | 1.72668 | 0.605549 |
| S17 | 236.715 | 0.997557 | 0.338204 | 2.54626 | 0.602465 |
| S18 | 185.599 | 0.998441 | 0.528002 | 3.87474 | 0.868876 |
| S19 | 103.364 | 0.998724 | 0.308476 | 1.9746 | 0.65215 |
| S20 | 451.402 | 0.993911 | 0.375885 | 3.14476 | 0.730917 |
| S21 | 289.567 | 0.996553 | 0.477679 | 3.67077 | 0.764487 |
| S22 | 205.589 | 0.99773 | 0.318538 | 2.33981 | 0.534962 |
| S23 | 238.875 | 0.997526 | 0.525026 | 3.98733 | 0.871859 |
| S24 | 313.187 | 0.996835 | 0.457992 | 3.65759 | 0.754722 |
| S25 | 198.747 | 0.997364 | 0.150362 | 1.06164 | 0.208634 |
| S26 | 185.657 | 0.998148 | 0.431437 | 3.08058 | 0.736213 |
| S27 | 259.84 | 0.99694 | 0.52946 | 4.03842 | 0.8471 |
| S28 | 323.428 | 0.995737 | 0.277552 | 2.18941 | 0.482458 |
| S29 | 173.135 | 0.998148 | 0.454713 | 3.21962 | 0.753532 |
| MT1 | 224.871 | 0.997766 | 0.484143 | 3.63448 | 0.804747 |
| MT2 | 260.113 | 0.996725 | 0.501759 | 3.6544 | 0.865808 |
| MT3 | 188.72 | 0.997918 | 0.202997 | 1.46365 | 0.286492 |
| MT4 | 199.498 | 0.997625 | 0.211562 | 1.48831 | 0.310139 |
| MT5 | 254.773 | 0.996626 | 0.243843 | 1.82567 | 0.374691 |
| MT6 | 360.168 | 0.995167 | 0.22207 | 1.78551 | 0.449025 |
| MT7 | 298.422 | 0.996542 | 0.58966 | 4.56971 | 0.91289 |
| MT8 | 284.536 | 0.996584 | 0.340159 | 2.61784 | 0.667068 |
| MT9 | 237.745 | 0.997071 | 0.187894 | 1.37669 | 0.264471 |
| MT10 | 140.413 | 0.998373 | 0.461953 | 3.10032 | 0.799502 |
| MT11 | 238.854 | 0.99762 | 0.354674 | 2.71207 | 0.625768 |
| MT12 | 227.366 | 0.997228 | 0.385144 | 2.84792 | 0.701445 |
| MT13 | 393.534 | 0.994623 | 0.407771 | 3.24396 | 0.753156 |
| MT14 | 119.292 | 0.998797 | 0.417563 | 2.68342 | 0.679837 |
| MT15 | 184.985 | 0.998169 | 0.556813 | 4.06961 | 0.884859 |
| MT16 | 189.775 | 0.998399 | 0.497971 | 3.60802 | 0.777909 |
| MT17 | 89.5167 | 0.999064 | 0.238016 | 1.48217 | 0.365487 |
| MT18 | 197.261 | 0.998201 | 0.348969 | 2.58259 | 0.519244 |
| MT19 | 129.496 | 0.998849 | 0.514431 | 3.46663 | 0.846462 |
| MT20 | 125.041 | 0.998399 | 0.406605 | 2.59851 | 0.730301 |
| MT21 | 357.881 | 0.996181 | 0.450105 | 3.64269 | 0.78732 |
| MT22 | 374.371 | 0.995705 | 0.32974 | 2.71105 | 0.668169 |
| MT23 | 255.928 | 0.997133 | 0.420597 | 3.21789 | 0.695367 |
| MT24 | 108.549 | 0.99875 | 0.364681 | 2.30676 | 0.704057 |
| MT25 | 161.601 | 0.998143 | 0.453531 | 3.06266 | 0.756854 |
| MT26 | 80.5768 | 0.999231 | 0.419826 | 2.53642 | 0.72904 |
| MT27 | 124.852 | 0.998797 | 0.410822 | 2.75583 | 0.728333 |
| MT28 | 81.1751 | 0.999132 | 0.303636 | 1.83363 | 0.600832 |
| MT29 | 235.362 | 0.997369 | 0.306179 | 2.31342 | 0.466107 |
| MT30 | 121.417 | 0.998797 | 0.438584 | 2.93503 | 0.676341 |
| LT1 | 150.909 | 0.997813 | 0.189045 | 1.27582 | 0.325683 |
| LT2 | 165.612 | 0.998211 | 0.506763 | 3.63126 | 0.847806 |
| LT3 | 106.988 | 0.99865 | 0.360608 | 2.25858 | 0.689831 |
| LT4 | 145.662 | 0.998227 | 0.287749 | 1.92557 | 0.50933 |
| LT5 | 61.7771 | 0.999262 | 0.253177 | 1.41359 | 0.501805 |
| LT6 | 67.6234 | 0.999132 | 0.099477 | 0.555765 | 0.148652 |
| LT7 | 158.425 | 0.998038 | 0.320072 | 2.14473 | 0.568243 |
| LT8 | 121.047 | 0.998928 | 0.455693 | 3.07481 | 0.779238 |
| LT9 | 65.0002 | 0.999106 | 0.187821 | 1.06457 | 0.316598 |
| LT10 | 152.746 | 0.997902 | 0.317331 | 2.16564 | 0.696139 |
| LT11 | 51.0583 | 0.999493 | 0.415518 | 2.14322 | 0.588218 |
| LT12 | 153.254 | 0.998274 | 0.361892 | 2.47537 | 0.683259 |
| LT13 | 128.44 | 0.998504 | 0.348165 | 2.31579 | 0.597238 |
| LT14 | 136.063 | 0.998269 | 0.250475 | 1.65169 | 0.423457 |
| LT15 | 53.0589 | 0.999404 | 0.358145 | 1.90802 | 0.691991 |

**Chao1** was used to estimate the richness of species in the community. **Good’s coverage** was used to estimate the proportion of OTUs without singletons in all OTUs. **Pielou’s evenness** was used to estimate the evenness of community. **Shannon** diversity and **Simpson** diversity were used to estimate community diversity.
