# Supplementary material for: Monitoring insect biodiversity and comparison of sampling strategies using metabarcoding: A case study in the Yanshan Mountains, China
Source: Ecol Evol. 2023 Apr 21;13(4):e10031. doi: 10.1002/ece3.10031 (PMC10121320; doi:10.1002/ece3.10031)
Supplement: Supplementary file 16 — Table S7 [file ECE3-13-e10031-s006.docx]

**Table S7 The exclusively detected families in the groups collected by different methods.**

|  | **sweep netting** | **Malaise traps** | **light traps** |  |  |
| --- | --- | --- | --- | --- | --- |
|  | Anaspis  Anthicidae  Erotylidae Laemophloeidae Monotomidae Oedemeridae Salpingidae Chamaemyiidae Lonchaeidae Aphididae Clastopteridae Flatidae  Largidae Membracidae Notonectidae Ortheziidae Plataspidae  Triozidae Urostylididae Figitidae Megalodontesidae Hesperiidae Lycaenidae  Pieridae  Gomphidae  Pyrgomorphidae Stenopsocidae | Psychodidae Nepticulidae Tischeriidae Psychidae Mutillidae Tropiduchidae Peripsocidae Hepialidae Cecidomyiidae  Limoniidae Scelionidae Crabronidae Autostichidae Coleophoridae Keroplatidae Byturidae Elachistidae Cosmopterigidae Fanniidae  Nolidae  Bethylidae Megaspilidae Eulophidae Silvanidae Platypezidae Gelechiidae Brentidae Megachilidae Baetidae Chrysididae Stratiomyidae Gryllacrididae Chalcididae Dryinidae Rhinophoridae Pediciidae Leiodidae Encyrtidae Milichiidae Zygaenidae Diapriidae Thripidae  Pythidae Lecithoceridae Pteromalidae | Cantharidae Lucanidae Silphidae Thespidae Leuctridae Lymantriidae Saturniidae Isonychiidae Leptophlebiidae Limacodidae Gerridae  Cydnidae Mycetophagidae Drepanidae Hydropsychidae Lasiocampidae Potamanthidae Scatopsidae Lepidostomatidae |  |  |
| Total | 27 | 45 | 19 |  |  |
