# Supplementary material for: Monitoring insect biodiversity and comparison of sampling strategies using metabarcoding: A case study in the Yanshan Mountains, China
Source: Ecol Evol. 2023 Apr 21;13(4):e10031. doi: 10.1002/ece3.10031 (PMC10121320; doi:10.1002/ece3.10031)
Supplement: Supplementary file 17 — Table S8 [file ECE3-13-e10031-s001.docx]

**Table S8 The exclusively detected families in the groups collected from different habitats.**

|  | **scrubland** | **wetland** | **woodland** | **farmland** | **grassland** |
| --- | --- | --- | --- | --- | --- |
|  | Thespidae Largidae Triozidae Membracidae Aeshnidae Urostylididae Latridiidae Gelechiidae Stenopsocidae Lasiocampidae Chalcididae Anaspis Lonchaeidae Encyrtidae Zygaenidae Aphididae Tineidae Erotylidae Ortheziidae Gomphidae Pteromalidae | Hesperiidae Chrysididae Rhinophoridae Leiodidae Milichiidae Megalodontesidae Lycaenidae | Diprionidae Peripsocidae Silphidae Leuctridae Chamaemyiidae Byturidae Laemophloeidae Leptophlebiidae Gerridae  Cydnidae Drepanidae Silvanidae Platypezidae Brentidae  Baetidae Stratiomyidae Gryllacrididae Figitidae Diapriidae Thripidae Clastopteridae Pythidae Lecithoceridae Salpingidae Lepidostomatidae | Coleophoridae Nolidae  Bethylidae Potamanthidae Monotomidae Pediciidae |  |
| Total | 21 | 7 | 25 | 6 | 0 |
