# Supplementary material for: Monitoring insect biodiversity and comparison of sampling strategies using metabarcoding: A case study in the Yanshan Mountains, China
Source: Ecol Evol. 2023 Apr 21;13(4):e10031. doi: 10.1002/ece3.10031 (PMC10121320; doi:10.1002/ece3.10031)
Supplement: Supplementary file 18 — Table S9 [file ECE3-13-e10031-s011.docx]

Table S9 Anosim tests for composition differences among different habitat categories.

| Group1 | Group2 | Sample size | Permutations | R | p value | q value |
| --- | --- | --- | --- | --- | --- | --- |
| all | - | 74 | 999 | 0.051102 | 0.078 | - |
| scrubland | wetland | 29 | 999 | 0.005624 | 0.45 | 0.5625 |
| scrubland | woodland | 52 | 999 | 0.016421 | 0.258 | 0.36857143 |
| scrubland | farmland | 38 | 999 | 0.053531 | 0.144 | 0.24 |
| scrubland | grassland | 27 | 999 | 0.192802 | 0.12 | 0.24 |
| wetland | woodland | 33 | 999 | -0.020803 | 0.531 | 0.59 |
| wetland | farmland | 19 | 999 | -0.052192 | 0.645 | 0.645 |
| wetland | grassland | 8 | 999 | 0.323077 | 0.074 | 0.24 |
| woodland | farmland | 42 | 999 | 0.060186 | 0.126 | 0.24 |
| woodland | grassland | 31 | 999 | 0.321835 | 0.031 | 0.195 |
| farmland | grassland | 17 | 999 | 0.305218 | 0.039 | 0.195 |
